# Predicting Post-Therapeutic Visual Acuity and OCT Images in Patients With Central Serous Chorioretinopathy by Artificial Intelligence

**DOI:** 10.3389/fbioe.2021.649221

**Published:** 2021-11-23

**Authors:** Fabao Xu, Cheng Wan, Lanqin Zhao, Shaopeng Liu, Jiaming Hong, Yifan Xiang, Qijing You, Lijun Zhou, Zhongwen Li, Songjian Gong, Yi Zhu, Chuan Chen, Li Zhang, Yajun Gong, Longhui Li, Cong Li, Xiayin Zhang, Chong Guo, Kunbei Lai, Chuangxin Huang, Daniel Ting, Haotian Lin, Chenjin Jin

**Affiliations:** ^1^ State Key Laboratory of Ophthalmology, Zhongshan Ophthalmic Center, Sun Yat-sen University, Guangzhou, China; ^2^ College of Electronic Information Engineering, Nanjing University of Aeronautics and Astronautics, Nanjing, China; ^3^ School of Computer Science, Guangdong Polytechnic Normal University, Guangzhou, China; ^4^ School of Medical Information Engineering, Guangzhou University of Chinese Medicine, Guangzhou, China; ^5^ Xiamen Eye Center, Affiliated with Xiamen University, Xiamen, China; ^6^ Department of Molecular and Cellular Pharmacology, University of Miami Miller School, Miami, FL, United States; ^7^ Department of Ophthalmology, The Central Hospital of Wuhan, Tongji Medical College, Huazhong University of Science and Technology, Wuhan, China; ^8^ Singapore National Eye Center, Department of Ophthalmology, Singapore, Singapore; ^9^ Center of Precision Medicine, Sun Yat-sen University, Guangzhou, China

**Keywords:** artificial intelligence, machine learning, central serous chorioretinopathy, visual acuity, optical coherence tomography

## Abstract

To predict visual acuity (VA) and post-therapeutic optical coherence tomography (OCT) images 1, 3, and 6 months after laser treatment in patients with central serous chorioretinopathy (CSC) by artificial intelligence (AI). Real-world clinical and imaging data were collected at Zhongshan Ophthalmic Center (ZOC) and Xiamen Eye Center (XEC). The data obtained from ZOC (416 eyes of 401 patients) were used as the training set; the data obtained from XEC (64 eyes of 60 patients) were used as the test set. Six different machine learning algorithms and a blending algorithm were used to predict VA, and a pix2pixHD method was adopted to predict post-therapeutic OCT images in patients after laser treatment. The data for VA predictions included clinical features obtained from electronic medical records (20 features) and measured features obtained from fundus fluorescein angiography, indocyanine green angiography, and OCT (145 features). The data for OCT predictions included 480 pairs of pre- and post-therapeutic OCT images. The VA and OCT images predicted by AI were compared with the ground truth. In the VA predictions of XEC dataset, the mean absolute errors (MAEs) were 0.074–0.098 logMAR (within four to five letters), and the root mean square errors were 0.096–0.127 logMAR (within five to seven letters) for the 1-, 3-, and 6-month predictions, respectively; in the post-therapeutic OCT predictions, only about 5.15% (5 of 97) of synthetic OCT images could be accurately identified as synthetic images. The MAEs of central macular thickness of synthetic OCT images were 30.15 ± 13.28 μm and 22.46 ± 9.71 μm for the 1- and 3-month predictions, respectively. This is the first study to apply AI to predict VA and post-therapeutic OCT of patients with CSC. This work establishes a reliable method of predicting prognosis 6 months in advance; the application of AI has the potential to help reduce patient anxiety and serve as a reference for ophthalmologists when choosing optimal laser treatments.

## Introduction

Central serous chorioretinopathy (CSC) is an idiopathic ophthalmopathy in which the neurosensory retina is often detached in the central macular region due to serous leakage from defects of the retinal pigment epithelium (RPE), causing damage to central vision (Saperstein et al., 2002; Piccolino et al., 2005; Maruko et al., 2010; Wong et al., 2016). Recurrent or persistent detachment is often associated with diffuse pathological RPE changes, which can be accompanied by secondary subretinal neovascularization and lead to permanent vision loss (Framme et al., 2015; Iacono et al., 2015; Manayath et al., 2018; Lee et al., 2019). Among nonsurgical retinopathies, CSC ranks fourth in incidence after age-related macular degeneration (AMD), diabetic retinopathy (DR), and retinal vein occlusion (RVO), and it is second only to AMD as the presumed cause of subretinal neovascularization (Wang et al., 2008; Manayath et al., 2018). Compared with AMD, DR, and RVO, which are generally seen in the elderly, CSC mostly affects young men of working age, exerting a heavy economic burden on families and society (Iacono et al., 2015; Daruich et al., 2017).

The increasing application of fundus fluorescein angiography (FFA), indocyanine green angiography (ICGA), optical coherence tomography angiography (OCTA), and optical coherence tomography (OCT) in CSC has greatly improved the understanding of its pathogenesis and provides an unprecedented opportunity to collect large-scale real-world imaging data (Feucht et al., 2016; Izumi et al., 2017; Cakir et al., 2019). Meanwhile, the application of artificial intelligence (AI) in the medical field is becoming increasingly popular (Caixinha and Nunes, 2017). The fundus photograph screening model of CSC and the subretinal fluid (SRF) segmentation model in OCT images developed by Zhen and Narendra et al. are both practical AI use cases (Narendra Rao et al., 2019; Zhen et al., 2019). AI is more widely used in screening for DR. Because of the increasing number of patients with DR and the lack of a sufficient number of ophthalmologists to perform screenings, efforts have been made to detect early forms of DR using AI. These AI programs show high efficiency and sensitivity (Oliveira et al., 2011; Haritoglou et al., 2014; Sim et al., 2015). In addition, previous studies have shown that AI can be applied to predict post-therapeutic visual acuity (VA) and OCT images based on automatic analysis of OCT imaging in patients with AMD (Bogunovic et al., 2017; Rohm et al., 2018; Liu et al., 2020).

A reliable prognostic prediction can help to alleviate emotional stress of patients with CSC. Personality traits and psychological disturbances are acknowledged as critical contributing factors in the development of CSC (Yannuzzi, 1987). Several studies have shown that psychological factors, typically anxiety, can trigger or exacerbate CSC (Yannuzzi, 1987; Spahn et al., 2003). In addition, an accurate prediction of VA based on different therapies as predictors can help ophthalmologists choose more appropriate and cost-effective treatment options. Therefore, the purposes of our study are to predict post-therapeutic VA and OCT images after different laser therapies in patients with CSC.

## Methods

### Clinical Data and Imaging Examinations

**FIGURE 1:**
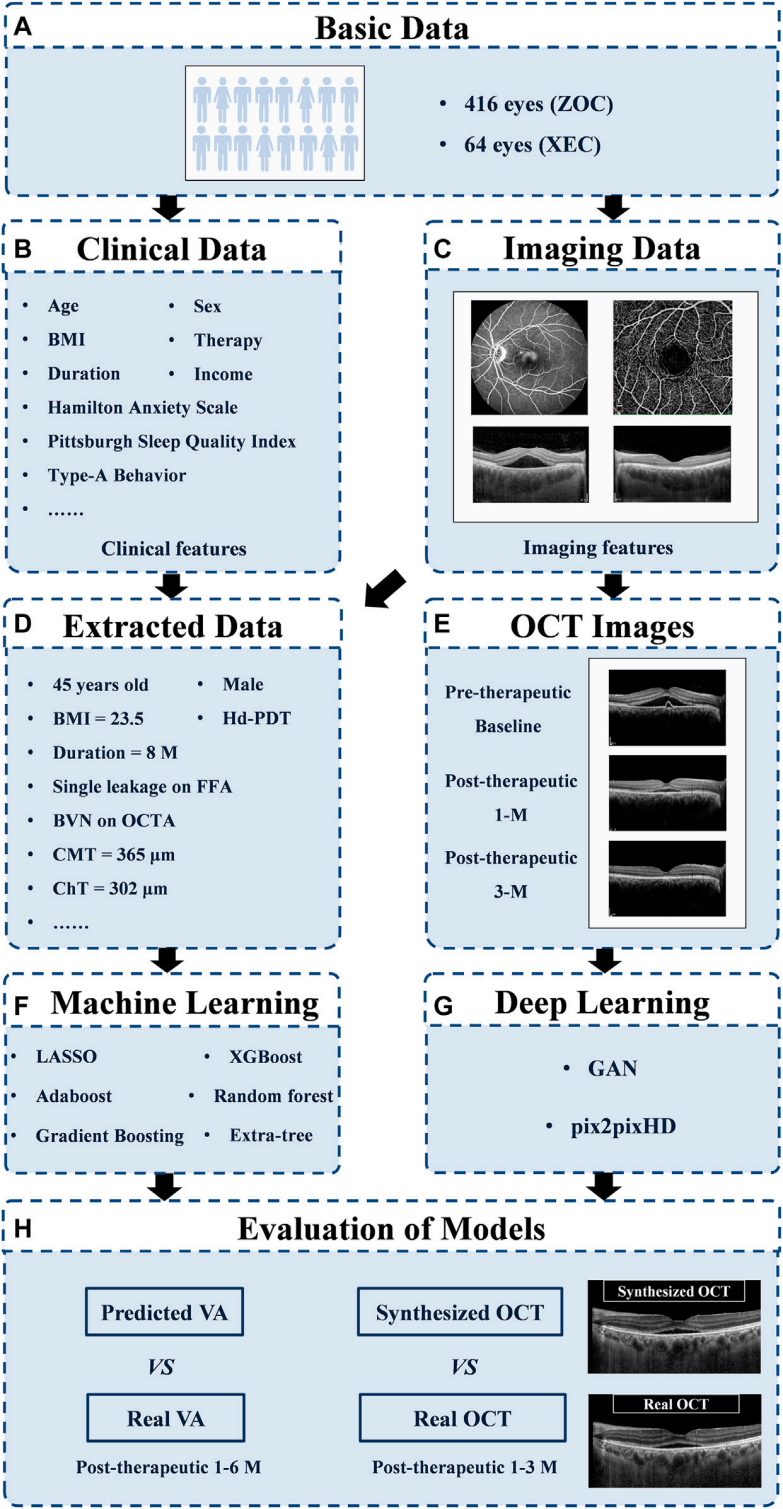
Overall study workflow. Workflow diagram showing the training overview for the CSC prognosis prediction model. ZOC, Zhongshan Ophthalmic Center; XEC, Xiamen Eye Center; OCT, optical coherence tomography; GAN, generative adversarial network; VA, visual acuity.

To estimate VA at 1, 3, and 6 months after laser treatment in patients with CSC, we applied machine learning algorithms to real-life data obtained from our data warehouse, including electronic medical records (20 clinical features, e.g., VA) and measured features from FFA, ICGA, OCTA, and OCT [145 features, e.g., the integrity of the ellipsoid zone (EZ); see Supplementary Figure S1 and Supplementary Table S1 for details]. To generate and evaluate individualized post-therapeutic OCT images that could predict the short-term response of laser therapies based on pre-therapeutic images using a generative adversarial network (GAN), a total of 416 pre- and post-therapeutic OCT images of patients with CSC obtained from Zhongshan Ophthalmic Center (ZOC) were included in the training set, whereas 64 pre-therapeutic OCT images obtained from Xiamen Eye Center (XEC) were included in the test set retrospectively, and the corresponding post-therapeutic OCT images were used to evaluate the synthetic images. The overall study workflow is shown in Figure 1. All the data collected from 1 January 2013 to 30 June 2019 included detailed information on 480 eyes of 461 CSC patients (416 eyes of 401 patients collected from ZOC, Sun Yat-sen University, were used as the training set; 64 eyes of 60 patients collected from XEC, affiliated with Xiamen University, were used as the test set). In our research, patients with CSC were given a definitive diagnosis on the basis of FFA and ICGA and underwent a comprehensive examination using OCTA and OCT. Only patients with SRF involving the fovea on OCT were considered for treatment. The exclusion criteria were as follows: 1) presence of any other chorioretinal diseases that may affect the study and 2) media opacities or an abnormal signal strength index on the OCT images. Regarding the laser therapies, the data from ZOC included conventional laser (CL) treatment (117 eyes), subthreshold micropulse laser (SML) treatment (80 eyes), and half-dose photodynamic therapy (hd-PDT) (219 eyes); the data from XEC included CL, SML, and hd-PDT treatment of 21, 14, and 29 eyes, respectively. In the case of persistent/increased SRF 3 months after the initial treatment or disease recurrence, the doctor decided whether to repeat or change the treatment on the basis of the multimodal images. The detailed protocol of the clinical flow is shown in the Supplementary Materials. The follow-up points were at 1, 3, and 6 months after the last laser treatment. However, it is difficult to perform follow-up visits with a fixed date because the patients with CSC were mostly young men with different work schedules. Thus, we determined a time range to ensure the accuracy of the study: 1 month ± 3 days; 3 months ± 5 days; and 6 months ± 7 days. Our ethics committee determined that written informed consent was not required because our study was retrospective in nature and all the images were fully anonymized. Moreover, this study adhered to the tenets of the Declaration of Helsinki (2020KYPJ024).

The ZOC data included 6,732 images (1,248 FFA, 1,248 ICGA, 1,412 OCTA, and 2,824 OCT), and the XEC data included 554 images (192 FFA and 362 OCT). FFA (Heidelberg Spectralis, Heidelberg, Germany), ICGA (Heidelberg Spectralis, Heidelberg, Germany), OCTA (RTVue XR Avanti with AngioVue; Optovue Inc., Fremont, CA, United States), and OCT (Heidelberg Spectralis, Heidelberg, Germany) measurements were extracted by a panel of 9 licensed Chinese retinal specialists (F. Xu, L. Zhou, Z. Li, Y. Xiang, L. Zhang, Y. Gong, L. Li, C. Li, and X. Zhang) and 2 senior professors (C. Jin and S. Gong) using the Heidelberg Eye Explorer (version 1.7.1.0) and Optovue (version 2017.1.0.155) software (Supplementary Figure S1 and Supplementary Table S1 ). All steps involving the training and testing were performed on a workstation with a 32-core Intel Xeon E5 CPU and 128 GB of RAM. We used Python (version 3.6.8) running under the Ubuntu 16.04 operating system, and the Python libraries used in this study are as follows: jupyter (1.1.0), scikit-learn (0.19.1), and pandas (0.20.3).

### Data Preprocessing of VA Predictions

The data from ZOC were used as the training and validation sets. For the 1-month VA prediction, a total of 401 patients (416 eyes) were included, and their mean VA at baseline, in terms of logarithm of the minimum angle of resolution (logMAR), was 0.28 ± 0.21. Among them, a total of 308 patients (322 eyes) had available follow-up data for the 3-month VA prediction, and a total of 244 patients (258 eyes) had sufficient follow-up data for the 6-month VA prediction. The data from XEC were used as the test set. For the 1-month VA prediction, 60 patients (64 eyes) were included, and the mean VA at baseline was 0.29 ± 0.16 logMAR. Among them, a total of 30 patients (33 eyes) had available follow-up data for the 3-month VA prediction, and a total of 19 patients (20 eyes) had sufficient follow-up data for the 6-month VA prediction.

During the data processing, we needed to preprocess the data and manage the missing values. Most of the machine learning models expects a dataset without any missing values; however, this is difficult to achieve in a real-life retrospective dataset. Therefore, we centered all the other values around OCT measurements. OCT was not missing in all patients and included follow-up visits. Because there were only a few missing values of ICGA features and questionnaire scales in the ZOC dataset, we used the mode and mean values of the corresponding features to fill in these missing qualitative and quantitative values. However, in the XEC dataset, large portions of the ICGA and OCTA features were not documented. Considering the applicability of the prediction models in the test set, we removed all missing features in the XEC dataset from the ZOC dataset in the training of the simplified models. For all of the above operations, we treated each eye as a separate case. After the preprocessing step, we used a total of 165 features to train each VA prediction algorithm in the full model.

### Algorithms Used for VA Predictions

To predict the logMAR VA of patients after laser treatment, we tested six regression algorithms with state-of-the-art performance in each adaptive domain. They are listed as follows: LASSO (Fujino et al., 2017), AdaBoost.R2 (Qi et al., 2018), Gradient Boosting (Luo et al., 2017), XGBoost (Ogunleye and Qing-Guo, 2019), Random Forest (Pavey et al., 2017), and Extra-Trees (Nattee et al., 2017) (see the Supplementary Materials for details). As seen by reviewing the websites of various algorithms from competitors (e.g., Kaggle), the algorithms that perform best in various projects are typically ensemble methods based on model combination approaches, such as stacking and blending (Baskin, 2018; Zhang et al., 2019). Therefore, we used the blending method to construct an ensemble of several different algorithms to obtain an algorithm with smaller bias and greater robustness. In our implementation, we first used the algorithms introduced above to train six regression models and then chose the best three to construct a new blending algorithm.

### Post-Therapeutic OCT Image Predictions

**FIGURE 2:**
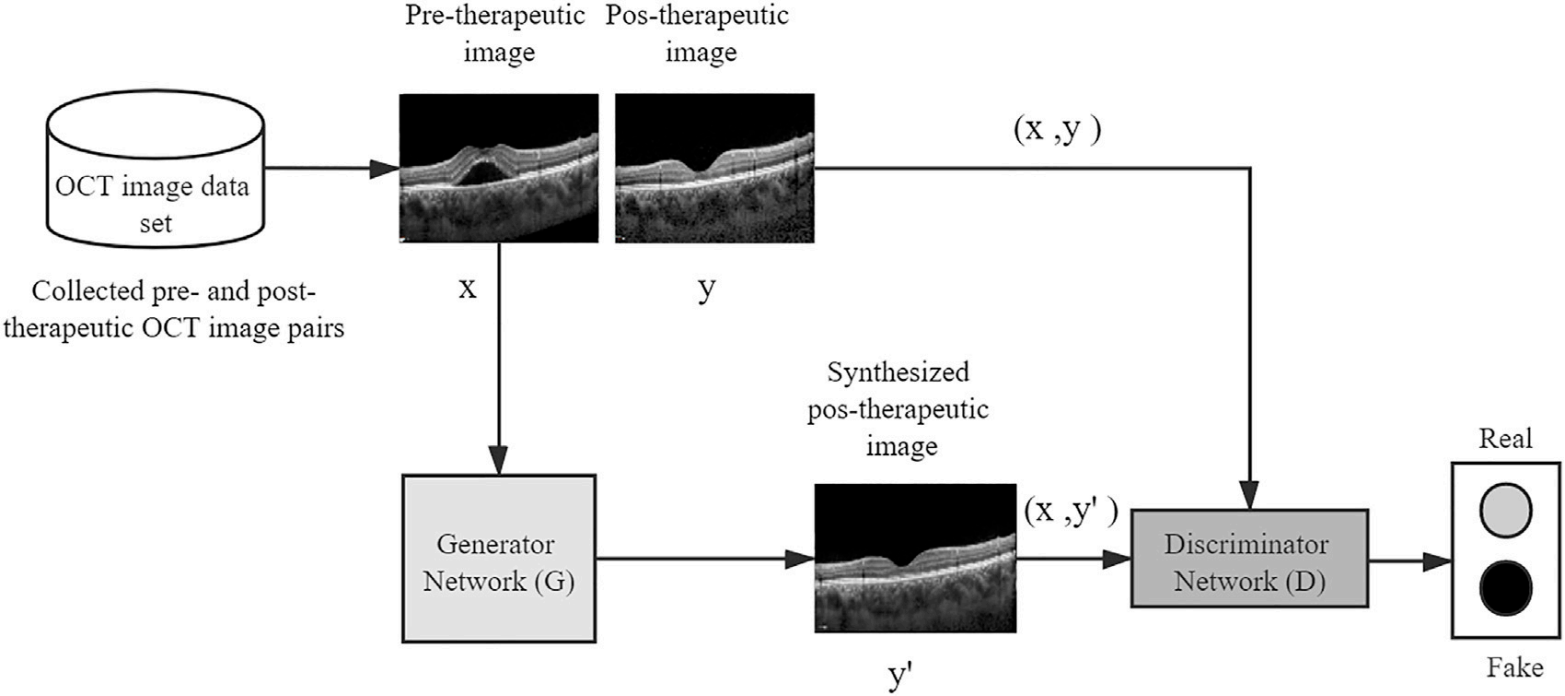
A conceptual illustration of generating post-therapeutic OCT from pre-therapeutic OCT by the pix2pixHD. OCT, optical coherence tomography.

To predict the post-therapeutic OCT image (1 and 3 months after laser therapy) using the pre-therapeutic OCT image, the pix2pixHD method was used for model training, which leveraged the conditional generative adversarial learning approach and fine-designed network architectures to achieve high visual synthesis performance. The pix2pixHD is a deep GAN-style algorithm, the training process of which is carried out in a game-playing manner. It consists of two different networks, namely, a discriminator network and a generator network. The generator network is a fine-designed network that aims to produce photo-realistic post-therapeutic images, whereas the discriminator network aims to discriminate real post-therapeutic images from the synthesized images. In the model training process, these two networks are first initialized from scratch and then update themselves iteratively like two competitive players in a game; specifically, the generator iteratively updates itself to produce images as close to real post-therapeutic images as possible, whereas the discriminator iteratively updates itself to identify the synthesized images as accurately as possible. After the training process is terminated, the generator network is able to translate any given pre-therapeutic OCT images to synthesized post-therapeutic images with high resolution (512 × 512 in our project). The framework of the entire model training process is depicted in Figure 2.

A total of 416 pairs of 1-month pre- and post-therapeutic OCT images and 322 pairs of 3-month pre- and post-therapeutic OCT images from 416 eyes with CSC were included in the training set, whereas 64 pairs of 1-month pre-therapeutic OCT images and 33 pairs of 3-month pre-therapeutic OCT images were included in the test set retrospectively, and their corresponding post-therapeutic OCT images were used to evaluate the synthetic images. A paired of OCT groups included the horizontal and vertical B scan pre- and post-therapeutic. The original OCT images had a resolution of 1,264 × 596 pixels. For every image included, the OCT B scan image with a size of 760 × 760 was cropped from the whole OCT image *via* contour recognition and zero-padding methods. All the cropped images were further resized to 512 × 512 pixels for model training.

### Development and Evaluation of VA Prediction Models

For the purpose of predicting the VA of CSC patients after laser treatment, we divided the prediction tasks into two types: the primary task was predicting VA with baseline data at 1, 3, and 6 months after laser treatment. For the secondary tasks, we further divided the prediction tasks into several categories according to the available data. We trained another model using the baseline and 1-month data for the task of predicting VA at 3 months after treatment. Similarly, we trained another two models: one using the baseline and 1-month data and the other one using the baseline and 1- and 3-month data, to predict VA at 6 months after treatment.

After completing the initial detailed investigation, we designed several simplified prediction models that would be more conveniently applied for clinical use. The first simplified prediction model, model Ⅰ, was trained with relatively few clinical data (eight clinical features, e.g., VA and duration) and all OCT features (Supplementary Table S2). The second simplified prediction model, model Ⅱ, was trained with VA and five types of OCT features [integrity of the EZ, central macular thickness (CMT), retinal neuroepithelial layer (RNEL), double-layer sign, and choroidal thickness]. Given the good predictive power of model Ⅱ, we removed VA and halved OCT data when training the third simplified prediction model, model Ⅲ. Therefore, model Ⅲ was trained with only five OCT features extracted from the horizontal B-scan used in model Ⅱ. Features were selected to be either dropped or retained in accordance with their relative importance as determined in the previous investigation and by the difficulty of image feature acquisition.

For all the VA prediction tasks mentioned above, we used 10-fold cross-validation to tune hyperparameters and assess the performance for each algorithm. We randomly divided the ZOC data into 10 subsets of nearly equal size, ensuring that the distribution of VA in each subset was similar to that in the original dataset. At each iteration, a training set consisted of nine subsets and was used to train a model, whereas the remaining subset was a validation set and was used to validate the performance of the model. The training and validation processes were repeated 10 times. The performance of an algorithm was assessed on the basis of the average performance achieved with the 10 validation datasets. First, hyperparameter tuning was carried out using a grid search with 10-fold cross-validation. Next, the optimal parameters were adopted to train the models. Then, the cross-validation performance of the six algorithms was compared, and the best three were selected to ensemble a new model by averaging the three predictions produced by them. In addition, the cross-validation performance of the ensembled model was calculated. Finally, the best three algorithms with their optimal parameters were trained on the entire ZOC dataset to generate the final ensembled model for feature importance interpretation and prediction. Then, we further assessed the performance of the ensemble models on the XEC dataset.

To quantitatively evaluate the performance of various algorithms, we applied the mean absolute error (MAE) and the root mean square error (RMSE) as the evaluation metrics. The MAE is calculated as the average value of the absolute error of the prediction results, which directly reflects the deviation of the predicted values from the actual values. The formula for the MAE is as follows:

$$\mathrm{MAE}=\frac{1}{N}\overset{N}{\underset{i=1}{\sum }}\left|{\tilde{y}}_{i}-y_{i}\right|$$

The RMSE is the square root of the mean square error (MSE), which has a greater penalty for samples with larger deviations. At the same time, because the RMSE and the original response variables are expressed in the same units, the RMSE is more interpretable than the MSE. The formula for the RMSE is as follows:

$$\mathrm{RMSE}=\sqrt{\frac{1}{N}\overset{N}{\underset{i=1}{\sum }}{\left({\tilde{y}}_{i}-y_{i}\right)}^{2}}$$

In the above two formulas, N is the number of predictions per fold, $\;y_{i}$ is the ground truth, and ${\tilde{y}}_{i}$ is the predicted value.

### Evaluation of Post-Therapeutic OCT Prediction Models

**FIGURE 3:**
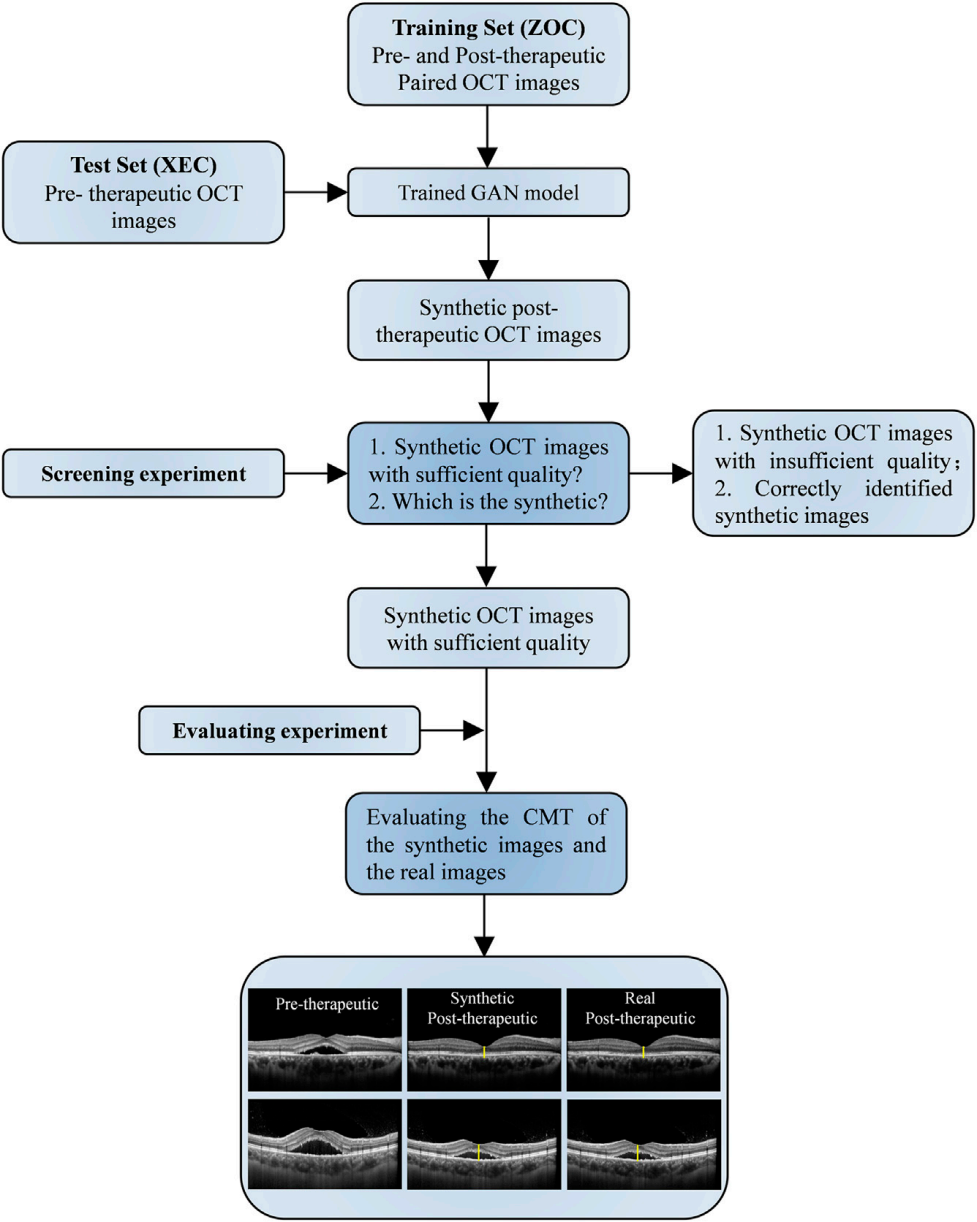
Workflow diagram of the synthetic OCT generation and evaluation. GAN, generative adversarial network; nAMD, neovascular age-related macular degeneration; OCT, optical coherence tomography.

Because the synthetic OCT images were supposed to be used for assisting clinical practice, the quality, similarity, and predictive power of the synthetic OCT images were evaluated by two experiments (Figure 3). Screening experiment refers to the evaluations of the quality and similarity of synthetic post-therapeutic OCT images. All synthetic images and paired real OCT images were marked and presented to two retinal specialists. They independently answered two questions: “Is the synthetic image of sufficient quality?” and “Can you identify the synthetic image, A, B, or undecided?” Only images that are difficult to distinguish from originals with sufficient quality were further analyzed in the evaluation experiment to determine whether the CMT of synthetic OCT is close to the real post-therapeutic OCT. We calculated the MAE of the CMT of the synthetic post-therapeutic OCT images. In addition, we also evaluated the residual SRF between the synthetic post-therapeutic OCT and the ground truth.

## Results

Descriptive data of the study population are provided in Table 1. The VA and post-therapeutic OCT images predicted by AI models were compared with the ground truth (Tables 2, 3).

**TABLE 1:** Patient demographics.

	1-month prediction		3-month prediction		6-month prediction	
	ZOC data	XEC data	ZOC data	XEC data	ZOC data	XEC data
Patients (Female)	401 (63)	60 (11)	308 (46)	30 (5)	244 (37)	19 (2)
Eyes	416	64	322	33	258	20
Hd-PDT/SML/CL	117/80/219	21/14/29	86/61/175	11/8/14	63/45/150	9/3/8
Age (Years)	43.19 ± 6.44	43.86 ± 7.06	42.87 ± 6.44	43.21 ± 7.51	42.96 ± 6.48	41.70 ± 6.73
VA (Baseline)	0.28 ± 0.21	0.29 ± 0.16	0.28 ± 0.21	0.27 ± 0.16	0.28 ± 0.22	0.28 ± 0.17
VA (Endpoint)	0.13 ± 0.16	0.11 ± 0.14	0.07 ± 0.17	0.07 ± 0.14	0.03 ± 0.17	0.04 ± 0.18

ZOC, Zhongshan Ophthalmic Center; XEC, Xiamen Eye Center; PDT, photodynamic therapy, half-dose PDT (hd-PDT) was applied in our study; SML, subthreshold micropulse laser treatment; CL, conventional laser treatment; VA, visual acuity, values are presented as the means ± standard deviations at baseline in different groups (in logarithm of minimum angle of resolution [logMAR] units).

**TABLE 2:** Accuracy of visual acuity predictions.

Algorithm learner	1 month		3 months		6 months	
Validation set	MAE	RMSE	MAE	RMSE	MAE	RMSE
Full model	0.057 ± 0.008	0.080 ± 0.011	0.070 ± 0.013	0.099 ± 0.021	0.072 ± 0.011	0.098 ± 0.019
Simplified model Ⅰ	0.057 ± 0.007	0.080 ± 0.010	0.071 ± 0.014	0.099 ± 0.023	0.073 ± 0.011	0.099 ± 0.018
Simplified model Ⅱ	0.058 ± 0.008	0.080 ± 0.010	0.072 ± 0.012	0.099 ± 0.019	0.075 ± 0.010	0.102 ± 0.016
Simplified model Ⅲ	0.077 ± 0.007	0.100 ± 0.010	0.079 ± 0.012	0.110 ± 0.022	0.080 ± 0.010	0.107 ± 0.014
**XEC set**	**MAE**	**RMSE**	**MAE**	**RMSE**	**MAE**	**RMSE**
Simplified model Ⅰ	0.074 (0.060–0.089)	0.096 (0.075–0.114)	0.074 (0.051–0.100)	0.103 (0.066–0.137)	0.092 (0.058–0.127)	0.121 (0.075–0.157)
Simplified model Ⅱ	0.074 (0.060–0.114)	0.096 (0.078–0.114)	0.073 (0.051–0.098)	0.101 (0.066–0.134)	0.094 (0.062–0.133)	0.123 (0.081–0.160)
Simplified model Ⅲ	0.083 (0.066–0.101)	0.110 (0.085–0.137)	0.089 (0.065–0.118)	0.119 (0.083–0.155)	0.098 (0.064–0.138)	0.127 (0.084–0.165)

MAE, mean absolute error; RMSE, root mean square error; XEC, Xiamen Eye Center. Accuracy (VA in logMAR) of VA prediction at 1, 3, and 6 months after laser treatment compared with the ground truth. The results were stratified according to the follow-up period and the points inputted into the algorithms; this table shows only the predictive effect of the baseline data. All VA predictions in the validation set are shown with the standard deviation (in logMAR); all VA predictions in the XEC set are shown with the 95% confidence interval.

**TABLE 3:** Accuracy of the synthetic post-therapeutic OCT images in determining the CMT and SRF in the evaluation experiment.

	Baseline	1-month prediction			3-month prediction		
CMT (μm)	Real images	Synthetic images	Real images	MAE (61 eyes)	Synthetic images	Real images	MAE (31 eyes)
Hd-PDT	333.29 ± 132.53	198.32 ± 64.12	189.06 ± 61.49	28.21 ± 12.34	194.54 ± 63.71	187.29 ± 61.02	19.36 ± 8.44
SML	375.41 ± 151.86	264.39 ± 79.58	254.86 ± 77.67	34.14 ± 14.56	254.41 ± 78.01	248.32 ± 77.77	28.65 ± 10.28
CL	334.23 ± 121.82	244.17 ± 95.47	234.69 ± 93.95	31.12 ± 13.52	230.24 ± 76.54	220.23 ± 76.21	21.44 ± 9.68
Total	357.83 ± 142.32	240.56 ± 83.28	228.24 ± 81.08	30.15 ± 13.28	231.45 ± 77.64	220.92 ± 75.76	22.46 ± 9.71
**SRF (μm)**	**Real images**	**Synthetic images**	**Real images**	**MAE**	**Synthetic images**	**Real images**	**MAE**
Hd-PDT	206.54 ± 119.56	77.84 ± 79.84	75.65 ± 78.15	19.21 ± 20.47	65.47 ± 79.87	63.26 ± 79.14	17.84 ± 20.17
SML	229.65 ± 135.11	86.77 ± 87.65	82.52 ± 82.44	24.26 ± 25.54	74.18 ± 89.74	71.25 ± 88.62	21.04 ± 23.83
CL	210.32 ± 117.48	80.19 ± 84.21	79.34 ± 80.89	22.16 ± 24.05	69.05 ± 86.92	67.94 ± 85.87	19.83 ± 22.86
Total	217.56 ± 130.36	81.57 ± 81.54	78.35 ± 80.42	22.44 ± 23.14	70.07 ± 87.19	68.04 ± 86.53	19.33 ± 22.08

CMT, central macular thickness; SRF, subretinal fluid; PDT, photodynamic therapy, half-dose PDT (hd-PDT) was applied in our clinical protocol; SML, subthreshold micropulse laser treatment; CL, conventional laser treatment; MAE, mean absolute error, values are presented as the means ± standard deviations.

In the comparative analysis of VA predictions, the blending algorithm exhibited the highest accuracy in VA prediction, outperforming the LASSO, AdaBoost, Gradient Boosting, XGBoost, Random Forest, and Extra-Trees models. Therefore, all subsequent analyses were conducted on the basis of the blending algorithm. In the full model, the MAEs of the VA predictions using baseline data with respect to the ground truth were 0.057 logMAR (within three letters), 0.070 logMAR (within four letters), and 0.072 logMAR (within four letters) for the 1-, 3-, and 6-month predictions, respectively, and the RMSEs of the VA predictions with respect to the ground truth were 0.080 logMAR (within four letters), 0.099 logMAR (within five letters), and 0.098 logMAR (within five letters) for the 1-, 3-, and 6-month predictions, respectively, using the 10-fold cross validation.

**FIGURE 4:**
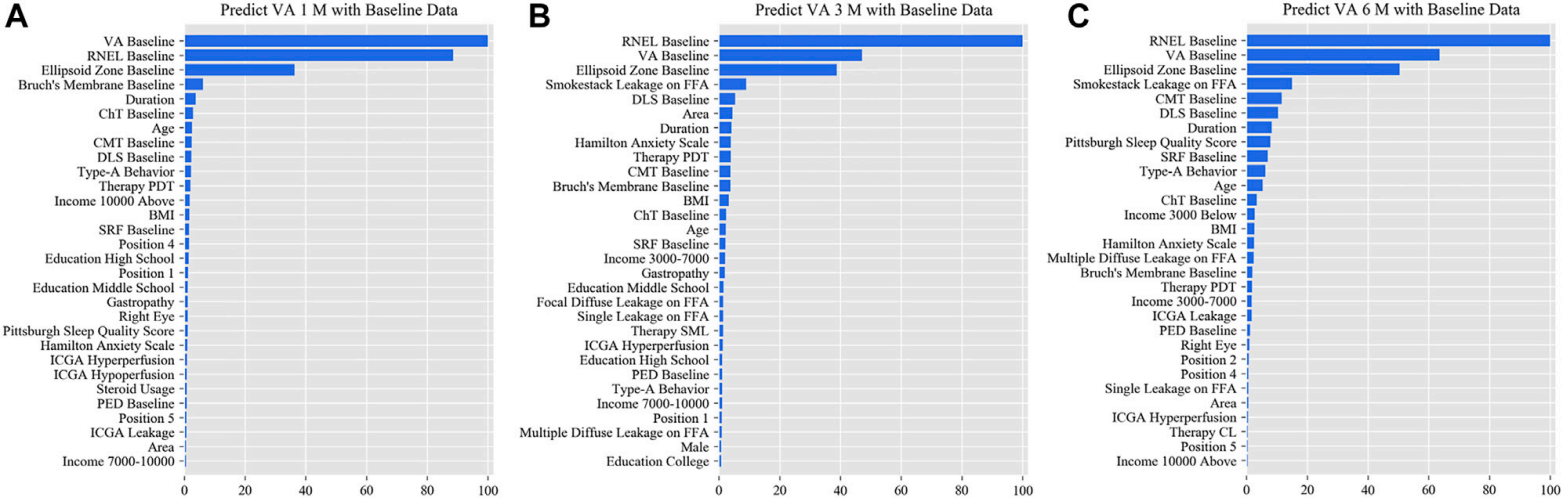
Relative importance of different features of prediction for the full model. These plots show the weights of the different features in the VA prediction task 1, 3, and 6 months after laser treatment in the full model. The results were stratified according to the follow-up period and the points input into the algorithms; this figure shows only the relative importance of the baseline data. The red bar indicates the average importance of the feature in the blending algorithm. **(A)** Feature weights in the 1-month VA prediction. **(B)** Feature weights in the 3-month VA prediction. **(C)** Feature weights in the 6-month VA prediction. VA, visual acuity; RNEL, retinal neuroepithelial layer; ChT, choroidal thickness; CMT, central macular thickness; DLS, double-layer sign; PDT, photodynamic therapy; SRF, subretinal fluid; ICGA, indocyanine green angiography; PED, pigment epithelial detachment.

**FIGURE 5:**
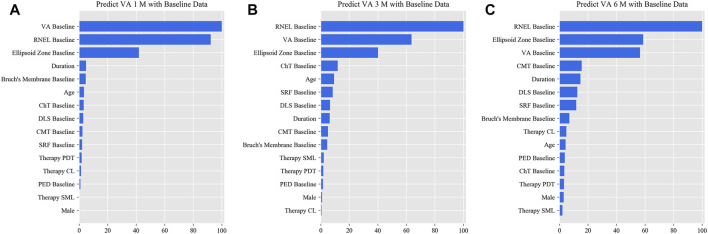
Relative importance of different features of prediction for simplified model Ⅰ. These plots show the weights of the different features in the VA prediction task 1, 3, and 6 months after laser treatment for model Ⅰ. The results were stratified according to the follow-up period and the points input into the algorithms; this figure shows only the relative importance of the baseline data. The red bar indicates the average importance of the feature in the blending algorithm. **(A)** Feature weights in the 1-month VA prediction. **(B)** Feature weights in the 3-month VA prediction. **(C)** Feature weights in the 6-month VA prediction.

**FIGURE 6:**
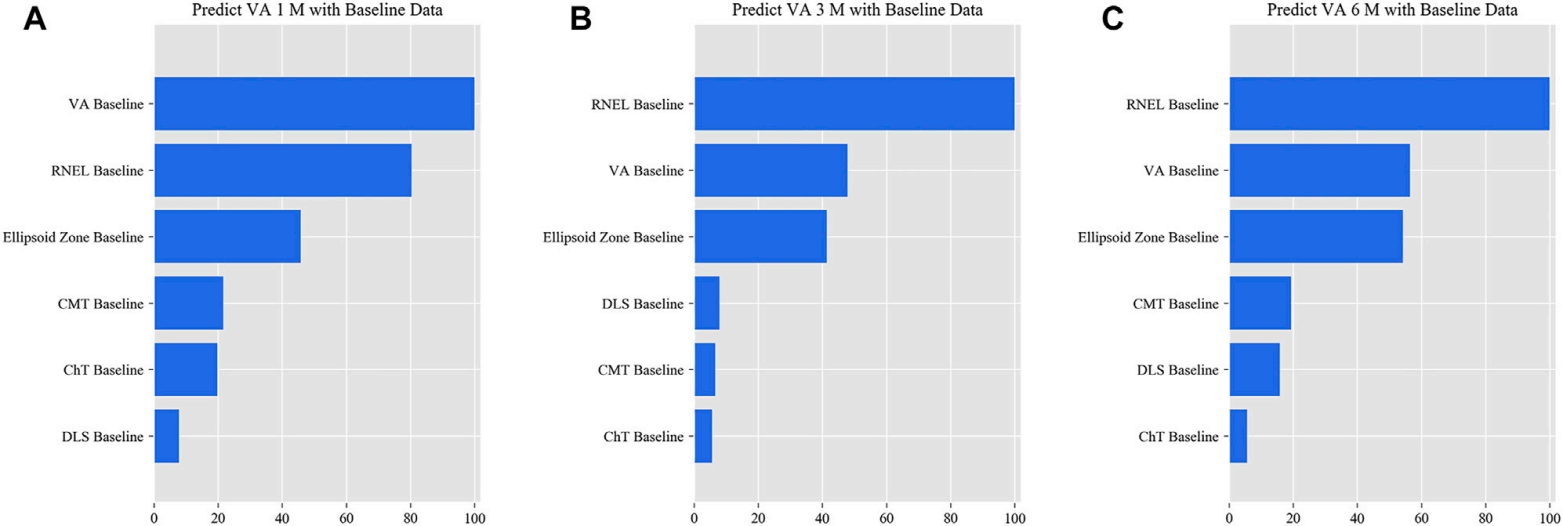
Relative importance of different features for prediction for simplified model Ⅱ. These plots show the weights of the different features in the VA prediction task 1, 3, and 6 months after laser treatment for model Ⅱ. The results were stratified according to the follow-up period and the points input into the algorithms; this figure shows only the relative importance of the baseline data. The red bar indicates the average importance of the feature in the blending algorithm. **(A)** Feature weights in the 1-month VA prediction. **(B)** Feature weights in the 3-month VA prediction. **(C)** Feature weights in the 6-month VA prediction.

**FIGURE 7:**
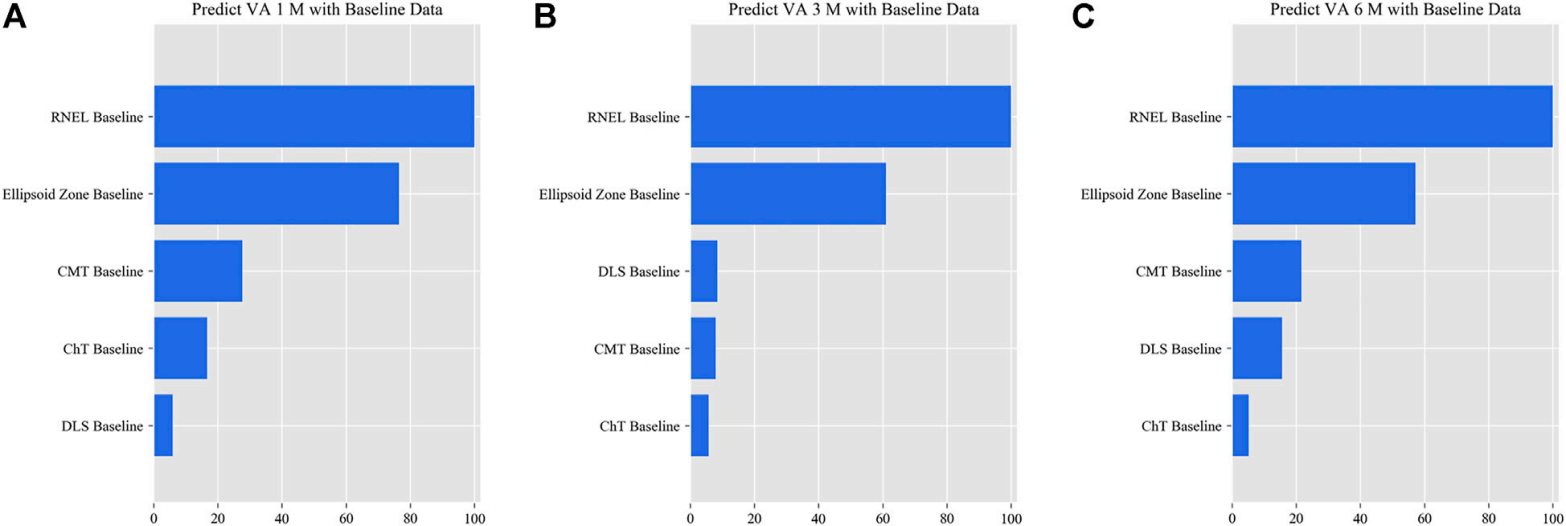
Relative importance of different features for prediction for simplified model Ⅲ. These plots show the weights of the different features in the VA prediction task 1, 3, and 6 months after laser treatment for model Ⅲ. The results were stratified according to the follow-up period and the points input into the algorithms; this figure shows only the relative importance of the baseline data. The red bar indicates the average importance of the feature in the blending algorithm. **(A)** Feature weights in the 1-month VA prediction. **(B)** Feature weights in the 3-month VA prediction. **(C)** Feature weights in the 6-month VA prediction.

In the simplified model Ⅰ, the MAEs of the VA predictions using baseline data, with respect to the ground truth, were 0.057 logMAR (within three letters), 0.071 logMAR (within four letters), and 0.073 logMAR (within four letters) for the 1-, 3-, and 6-month predictions, respectively, and the RMSEs of the VA predictions with respect to the ground truth were 0.080 logMAR (within four letters), 0.099 logMAR (within five letters), and 0.098 logMAR (within five letters) for the 1-, 3-, and 6-month predictions, respectively, using the validation set. In the test set, the MAEs of the VA predictions with respect to the ground truth were 0.074 logMAR (within four letters), 0.074 logMAR (within four letters), and 0.091 logMAR (within five letters) for the 1-, 3-, and 6-month predictions, respectively, and the RMSEs of the VA predictions with respect to the ground truth were 0.096 logMAR (within five letters), 0.103 logMAR (within six letters), and 0.119 logMAR (within six letters) for the 1-, 3-, and 6-month predictions, respectively. The second simplified model, model Ⅱ, achieved a comparable level of predictive power with fewer features, and although the accuracy of model Ⅲ declined slightly, the error remained within seven letters overall in terms of MAEs and RMSEs. When the previous follow-up data were considered in the four models, the performances for long-term predictions were improved compared with those achieved using the baseline data alone (Supplementary Tables S3–S6). Supplementary Figures S2–S4 show the differences between the predicted and ground-truth VA values, which were the VA values measured at XEC. Figures 4–7 show the relative importance of the features for the VA predictions.

**FIGURE 8:**
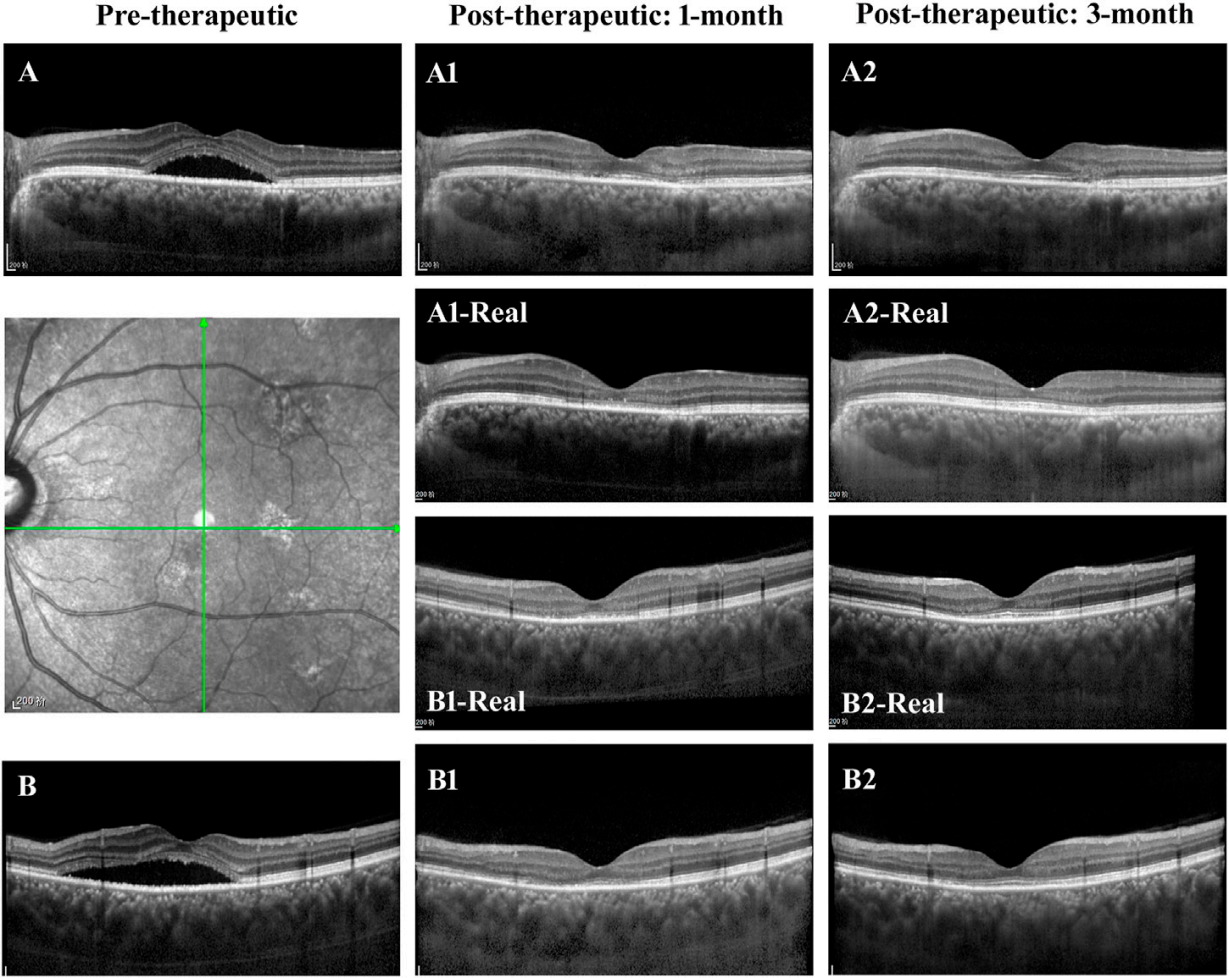
Illustrations of the synthetic OCT of the GAN model. **(A**,**B)** The horizontal and vertical OCT B scans of a patient with CSC. **(A1**,**A2**,**B1**,**B2)** The synthetic post-therapeutic images generated by the pix2pixHD. The images in the middle are the corresponding real images.

In the evaluation of post-therapeutic OCT predictions, a total of 64 pairs of 1-month and 33 pairs of 3-month synthetic post-therapeutic images were generated on the basis of the pre-therapeutic OCT images of patients with CSC (Figure 8). In the screening experiment, the two retinal specialists (C. Jin and S. Gong) disagreed on the judgment of one pair of 1-month synthetic images; specialist 1 considered it unqualified, whereas specialist 2 considered it qualified. Finally, the third specialist (D. Ting) was consulted, and the images were deemed to be qualified. In the experiment performed to distinguish the synthetic OCT images from the real images, specialist 1 accurately identified three pairs of 1-month synthetic and two pairs of 3-month synthetic images, and specialist 2 accurately identified two pairs of 1-month synthetic and two pairs of 3-month synthetic images. Most of the images (95.31% at 1 month and 93.94% at 3 months) were judged to be indistinguishable by the specialists. All the synthetic images that could not be identified were analyzed in the evaluation experiment.

In the evaluation experiment, the two retinal specialists measured the CMT and the SRF of all the synthetic post-therapeutic OCT images. No significant difference was found between the two datasets on the basis of the Pearson correlation (Pearson values > 0.8). The mean values between the two measurements were used for further analysis. The MAEs of CMT and SRF between the synthetic OCT images and real images are shown in Table 3 in detail.

## Discussion

This is the first study to predict post-therapeutic VA and OCT images in CSC patients by AI. We demonstrated that post-therapeutic VA and OCT images can be accurately predicted within a very small error using machine learning and deep learning algorithms on the basis of five models. In the VA predictions, the first and second simplified models, models Ⅰ and Ⅱ, achieved a level of predictive power comparable with that of the full model in terms of MAEs and exhibited only a slight decline in predictive power in terms of RMSEs. The short-term predictive power of model Ⅲ declined slightly compared with that of the previous three models, but the prediction errors could still be controlled within one or two lines on the vision chart after 6 months. For patients with CSC, the most significant predictor of short-term VA was the most recent VA measurement; however, for the long-term VA predictions, the retinal integrity, such as RNEL, is more important (Figures 4–7). In the post-therapeutic OCT predictions, more than 90% of the generated images could not be distinguished by retinal specialists. The MAEs of the CMT and the SRF between synthetic OCT images and real OCT images were approximately 30 and 20 μm at the 1- and 3-month predictions, respectively.

The primary concern of patients with CSC is the restoration of VA (Wang et al., 2008; Wong et al., 2016). In most fundus disease clinical trials, improving VA is the primary goal, as it not only has scientific value but also provides an indication of how patients are affected by the disease. Reliable VA predictions reassure patients before treatment and alleviate their emotional distress, which may play a positive role in recovery from CSC (Yannuzzi, 1987; Wang et al., 2008). In addition, VA predictions may encourage patients to cooperate with treatment and comply with follow-up. As indicated by the fitting curves for VA predictions, short-term predictions tend to outperform long-term predictions, and more follow-up points are likely to improve prediction accuracy (Supplementary Tables S3–S6). Moreover, the predictions of post-therapeutic OCT images can be used as a supplement to VA predictions. Accurate structural prediction allows the doctors to foresee the therapeutic effect on OCT more intuitively. However, given the small number of paired OCT images at 6 months after laser therapies and the possibility of recurrence, our predictions for post-therapeutic OCT were limited to the third month.

Interestingly, the simplified VA prediction models were comparable with the full model in terms of performance, and all the individual algorithms achieved good performance. This observation implies that feature extraction was crucial for achieving high accuracy with machine learning models, whereas algorithms based on different principles did not lead to much difference in the results. In general, features need to be collected as comprehensively as possible during the data collection phase of a machine learning study to improve prediction accuracy (Baskin, 2018; Zhang et al., 2019). The full model is the basis of simplified models and exhibits the greatest potential performance that we can achieve. However, exhaustive feature collection is laborious, especially in real clinical settings. In addition, it is not feasible to perform higher-dimensional examinations in patients to satisfy the requirements of AI, considering the invasive nature of angiography and the unavailability of OCTA facilities in many underdeveloped regions. Fortunately, our simplified models have achieved good predictive performance, enabling the prediction of VA in patients after laser treatment 6 months in advance based on OCT images. This success greatly expands the potential application scenarios of our study.

Notably, however, the clinical application of VA prediction models should be highly individualized. Currently, laser therapies, including CL treatment, SML treatment, and hd-PDT, have shown great effectiveness and safety in various clinical studies (Burumcek et al., 1997; Lim et al., 2011; Scholz et al., 2016; Zhou et al., 2019). However, clinical data collected in the real world are often subject to bias due to the rigorous entry criteria of most clinical studies. In addition, disturbances related to refractory and repeatedly recurrent CSC are often masked by the average efficiency of most CSC clinical studies (Doepfner et al., 2018; Sartini et al., 2019). Moreover, the improvement observed in the average VA after treatment does not represent the final improvement in vision for every CSC patient even if the SRF has been completely absorbed, as EZ atrophy that has occurred over a long time is not easily recovered in the short term (Hasegawa et al., 2015; Chung et al., 2018; van Rijssen et al., 2018). In this study, we comprehensively measured the clinical features that might affect the prognosis of CSC patients in the full model and then identified the predictors that substantially affected VA restoration. Detailed feature selection and alternative models that accounted for the complexity of realistic clinical environments could potentially increase the robustness of our predictions.

Clinical and imaging features are predictors of VA; conversely, VA predictions also reveal the factors that influence VA prognosis. Our study offers a novel approach for identifying essential factors that influence VA restoration. As shown in our study, the features of the retina at baseline, such as the EZ, RNEL, and CMT, are critical for VA prognosis; by comparison, the choice of CL treatment, SML treatment or hd-PDT is less important. This method of solving clinical problems is different from those of clinical trials that compare the effectiveness of different laser therapies, such as the PLACE study in 2018, which concluded that hd-PDT was superior to SML for treating chronic CSC (van Dijk et al., 2018). However, this conclusion has been reached for a scenario with limited inclusion criteria and may not be applicable in acute CSC and other clinical scenarios. Therefore, the main importance of our study is serving as a more holistic reference to help ophthalmologists choose more cost-effective laser therapy for patients with CSC, as hd-PDT requires patients to bear a high cost, whereas CL and SML treatment are much less expensive. To be more specific, we can change the therapy that we enter into the model to calculate the improvement in VA before the operation. Therefore, an important contribution of our study is the provision of a more holistic reference to help ophthalmologists choose the most cost-effective laser therapy option for patients with CSC, as CL and SML treatment are much less expensive than hd-PDT.

The present study has some limitations. First, more and longer follow-up data are still necessary to improve the accuracy and stability of the post-therapeutic VA and OCT prediction models. Second, extracting features manually in machine learning is a very labor-intensive and time-consuming process. With additional data, we will likely be able to use more computationally intensive approaches like deep learning to achieve VA predictions. Finally, observation is one of the most important managements due to the self-limiting of patients with CSC; however, our study did not enroll follow-up data of observed patients.

In summary, our study shows that multidimensional patterns in clinical and imaging data can be used as predictive factors for post-therapeutic VA and OCT predictions in CSC patients. The proposed models are the first to enable personalized, objective, and reproducible prediction of the therapeutic effect. This work presents a novel direction of medical data mining to support clinical practice and guide precise individualized interventions.

## Data Availability

The raw data supporting the conclusions of this article will be made available by the authors, without undue reservation.
