# Supplementary material for: Predicting Post-Therapeutic Visual Acuity and OCT Images in Patients With Central Serous Chorioretinopathy by Artificial Intelligence
Source: Front Bioeng Biotechnol. 2021 Nov 23;9:649221. doi: 10.3389/fbioe.2021.649221 (PMC8650495; doi:10.3389/fbioe.2021.649221)
Supplement: Supplementary file 1 [file DataSheet1.docx]

Supplemental Materials

Flowchart of the proposed treatment strategy 2

[Six separated regression algorithms 6](#_Toc30900043)

[LASSO](#_Toc30900044) **[6](#_Toc30900044)**

[AdaBoost.R2 **6**](#_Toc30900045)

[Gradient Boosting **6**](#_Toc30900046)

[XGBoost **6**](#_Toc30900047)

[Random Forest **7**](#_Toc30900048)

[Extra-Trees **7**](#_Toc30900049)

[Table S1 8](#_Toc30900050)

[Table S2 10](#_Toc30900051)

[Table S3 12](#_Toc30900051)

[Table S4 13](#_Toc30900051)

[Table S5 1](#_Toc30900051)4

[Table S6 15](#_Toc30900051)

[Figure S1 16](#_Toc30900052)

[Figure S2 17](#_Toc30900052)

[Figure S3 18](#_Toc30900052)

[Figure S4 18](#_Toc30900052)

**Flowchart of the proposed treatment strategy:**

**Acute CSC**

1. First CSC-related visual symptoms for longer than 2-4 months
2. Presence of SRF
3. One or more active leakages on FFA
4. No signs of chronic or complex CSC (e.g., atrophic RPE changes; cystoid intraretinal changes; CNV on OCTA)

**One of the following conditions**

1. Highly symptomatic
2. Recurrent episode
3. Bilateral
4. Patient preference for treatment
5. SRF involving the fovea on OCT

**No**

**Yes**

**Observation for 1-3 months after onset of the first episode**

**One of the following options**

1. Conventional laser (in cases of active leakage away from the fovea)
2. Subthreshold micropulse laser
3. FFA- and ICGA-guided half-dose PDT^1^

**No SRF on OCT**

**Persistent SRF on OCT**^2^

**Persistent SRF on OCT after 3 months**^2^

**No SRF on OCT**

**Obtain OCTA, FFA, and ICGA**

**No leakage/hyperfluorescence on FFA/ICGA, no CNV**

**Persistent focal or diffuse leakage on FFA/ICGA without evidence of other diagnoses**

**Presence of CNV**

**No solid evidence for effective treatment (retreatment)**

**Intravitreal anti-VEGF treatment (****or with PDT**^3^**)**

**Retreatment guided by leakage on FFA/ICGA**^4^ **(or half-dose PDT after 6 months)**

Flowchart of the proposed treatment strategy for acute CSC. If there is current corticosteroid use, possible cessation should be discussed with the patient.

^1^ Treat hyperﬂuorescent areas on indocyanine green angiography (ICGA) that correspond to the area of (focal) leakage on FFA and subretinal ﬂuid on OCT. In the case of multiple areas with focal leakage, a large spot including all areas can be used, or multifocal immediately sequential spots may be used, starting with the area including the fovea.

^2^ In the case of only a small amount of residual subretinal ﬂuid (SRF), a conservative approach may be followed, with a follow-up visit including OCT imaging after 1–3 months to see if SRF eventually resolves completely. In the case of persistent/increased SRF at that stage, the downstream treatment path may be followed.

^3^ Half-dose PDT may be added in order to treat the choroidal dysfunction/pachychoroid factor of the disease, but limited data are available to support this combined treatment. When a neovascular component of polypoidal choroidal vasculopathy (aneurysmal type 1 neovascularization) is present, PDT (either full-dose, half-dose, or half-ﬂuence) can also be added to anti-VEGF.

^4^ The second treatment is usually the same as the initial treatment, and half-dose PDT can be performed if the SRF increases or for longer more than 6 months.

**Chronic CSC**

1. Persistent SRF on OCT for longer than 6 months
2. Atrophic RPE changes
3. One or more active leakages on FFA
4. Hyperfluorescent choroidal abnormalities on ICGA
5. No signs of CNV or other macular diseases on OCTA

**One of the following options**

1. FFA- and ICGA-guided half-dose PDT^1^
2. Conventional laser (in cases of active leakage away from the fovea)
3. Subthreshold micropulse laser

**Persistent SRF on OCT after 3 months**^2^

**No SRF on OCT**

**Obtain OCTA, FFA, and ICGA**

**No leakage/hyperfluorescence on FFA/ICGA, no CNV**

**Presence of CNV**

**Persistent focal or diffuse leakage on FFA/ICGA without evidence of other diagnoses**

**No solid evidence for effective treatment (retreatment)**

**Intravitreal anti-VEGF treatment (or with PDT**^3^**)**

**Retreatment guided by leakage on FFA/ICGA**^4^ **(or half-dose PDT after 6 months)**

Flowchart of the proposed treatment strategy for chronic CSC. If there is current corticosteroid use, possible cessation should be discussed with the patient.

^1^ Treat hyperﬂuorescent areas on indocyanine green angiography (ICGA) that correspond to the area of (focal) leakage on FFA and subretinal ﬂuid on OCT. In the case of multiple areas with focal leakage, a large spot including all areas can be used, or multifocal immediately sequential spots may be used, starting with the area including the fovea. In chronic CSC, the most commonly recommended treatment is half-dose PDT. However, there are still some chronic CSC patients who can consider other treatments.

^2^ In the case of only a small amount of residual subretinal ﬂuid (SRF), a conservative approach may be followed, with a follow-up visit including OCT imaging after 1–3 months to determine whether SRF eventually resolves completely. In the case of persistent/increased SRF at that stage, the downstream treatment path may be followed.

^3^ Half-dose PDT may be added in order to treat the choroidal dysfunction/pachychoroid factor of the disease, but limited data are available to support this combined treatment. When a neovascular component of polypoidal choroidal vasculopathy (aneurysmal type 1 neovascularization) is present, PDT (either full-dose, half-dose, or half-ﬂuence) can also be added to anti-VEGF.

^4^ The second treatment is usually the same as the initial treatment, and half-dose PDT can be performed if the SRF increases or persists longer than 6 months after the initial therapy.

**Abbreviations in the ﬂowchart:** CNV, choroidal neovascularization; CSC, central serous chorioretinopathy; FFA, fundus ﬂuorescein angiography; ICGA, indocyanine green angiography; OCT, optical coherence tomography; OCTA, optical coherence tomography angiography; PDT, photodynamic therapy; RPE, retinal pigment epithelium; SRF, subretinal ﬂuid; VEGF, vascular endothelial growth factor.

**The six separate regression algorithms used for visual acuity prediction are listed as follows:**

**LASSO:** The least absolute shrinkage and selection operator (LASSO) is a linear model that estimates sparse coefficients using an L1 regularization technique. It achieves the shrinkage and selection of variables while estimating parameters and more effectively solves the multicollinearity problem in regression analysis. Because it tends to select solutions with fewer nonzero coefficients, LASSO is often used for feature selection.

**AdaBoost.R2:** AdaBoost is a boosting algorithm based on the idea of fitting a sequence of weak learners by iterating over the same training set, and the final prediction results are obtained by calculating the weighted combination of the outputs of these weak learners. In the implementation of boosting algorithms, the weight of a sample with poor performance in the previous learner is increased, and the updated sample is then used to retrain the next weak learner. When combining all learners, the weight of each weak learner is determined based on its performance.

**Gradient Boosting:** Gradient Boosting is a generalization of boosting to arbitrary differentiable loss functions. In this method, the negative gradient of the loss function (the first derivative of the loss function) is used as a measure of the performance of a weak learner, and the weak learner is optimized by reducing the loss function in the direction of the gradient.

**XGBoost:** Extreme gradient boosting (XGBoost) is an optimized distributed gradient boosting algorithm implemented based on the original gradient boosting framework. Instead of the first derivative, which is used in gradient boosting, the first-order and second-order Taylor expansions of the loss function are used in the optimization process in XGBoost. Consequently, its accuracy is better, and fewer iterations are required to achieve satisfactory results. Unlike other boosting methods, XGBoost allows the use of multithreading when choosing the best segmentation point. The parallel tree boosting operation substantially reduces the run time.

**Random Forest:** The random forest algorithm is a variant of the bagging (Bootstrap AGGregatING) algorithm that obtains its final results by averaging the predictions of many decision trees. The training set used to construct each decision tree is obtained using the bootstrap method (random sampling with replacement from the original data). Furthermore, when splitting one node during the construction of a tree, a subset of all features at that node is randomly selected, and an optimal feature is then selected from this subset for splitting. Because of the use of random sampling and random feature selection, the random forest algorithm is not easily susceptible to overfitting, although no pruning is performed on any single tree.

**Extra-Trees:** The Extremely Randomized Trees (Extra-Trees) algorithm is a variant of the random forest algorithm obtained by introducing random thresholds when splitting nodes. The random forest algorithm uses the bootstrap method to obtain the training set, whereas Extra-Trees uses all samples for training. Instead of choosing the most discriminative thresholds in feature subsets, as in the random forest algorithm, Extra-Trees randomly selects thresholds for the candidate features and then selects the best threshold for node splitting.

| **Clinical data** | | | | **Features from FFA and ICGA** | | **Features from OCTA** | |
| --- | --- | --- | --- | --- | --- | --- | --- |
| **Feature** | **Description** | **Feature** | **Description** | **Feature** | **Description** | **Feature** | **Description** |
| Age  Sex  Height  Weight  Education  Income  Heart Disease  Gastropathy  Autoimmune Disease  Steroid Usage  Hamilton Anxiety Scale  Pittsburgh Sleep Quality Index | Age of the patient  Sex of the patient  Height of the patient  Weight of the patient  Education level of the patient  Income level of the patient  History of heart disease  History of gastropathy  History of autoimmune disease  History of steroid use  Hamilton Anxiety Scale^1^ score  Pittsburgh Sleep Quality Index^2^ score | Type-A Behavior  Eye  Duration  Therapy  VA Baseline  VA 1-mo  VA 3-mo  VA 6-mo | Type-A Behavior^3^ score  Right or left eye  Duration of CSC  hd-PDT, SML or CL  VA before treatment  VA at 1-mo after treatment  VA at 3-mo after treatment  VA at 6-mo after treatment | FFA leakage  Single or multiple  Morphology  Area  Position 1  ICGA leakage  High permeability  Position 2  Low permeability  Position 3 | Existence of active leakage at baseline  No. of active leakage sites on FFA  Morphology of the leakage on FFA  Area of the leakage on FFA  Position of the leakage on FFA  Existence of active leakage at baseline  High permeability on ICGA  Position of the high permeability on ICGA  Low permeability on ICGA  Position of the low permeability on ICGA | High reflection  Position 4  Low reflection  Position 5  BVN Baseline  Position 6  BVN 1-mo  Position 7  BVN 3-mo  Position 8  BVN 6-mo  Position 9 | Existence of high reflection at baseline  Position of high reflection on OCTA  Existence of low reflection at baseline  Position of low reflection on OCTA  Existence of BVN at baseline  Position of BVN at baseline  Existence of BVN at 1-mo  Position of BVN at 1-mo  Existence of BVN at 3-mo  Position of BVN at 3-mo  Existence of BVN at 6-mo  Position of BVN at 6-mo |
| **Features from OCT (Baseline)** | | **Features from OCT (1-mo)** | | **Features from OCT (3-mo)** | | **Features from OCT (6-mo)** | |
| **Feature** | **Description** | **Feature** | **Description** | **Feature** | **Description** | **Feature** | **Description** |
| SFA horizontal  SFA vertical  SFA  CMT horizontal  CMT vertical  CMT  RNEL horizontal  RNEL vertical  RNEL  SRF horizontal  SRF vertical  SRF  ChT horizontal  ChT vertical  ChT  EZ horizontal  EZ vertical  EZ  PED horizontal  PED vertical  PED  DLS horizontal  DLS vertical  DLS  Bruch’s membrane horizontal  Bruch’s membrane vertical  Bruch’s membrane | Subretinal fluid absorption on horizontal B-scan  Subretinal fluid absorption on vertical B-scan  Subretinal fluid absorption at baseline  CMT on horizontal B-scan  CMT on vertical B-scan  Average CMT at baseline  Thickness of RNEL on horizontal B-scan  Thickness of RNEL on vertical B-scan  Average thickness of RNEL at baseline  Height of SRF on horizontal B-scan  Height of SRF on vertical B-scan  Average height of SRF at baseline  ChT on horizontal B-scan  ChT on vertical B-scan  Average ChT at baseline  Integrity of EZ on horizontal B-scan  Integrity of EZ on vertical B-scan  Average integrity of EZ at baseline  Existence of PED on horizontal B-scan  Existence of PED on vertical B-scan  Existence of PED at baseline  Existence of DLS on horizontal B-scan  Existence of DLS on vertical B-scan  Existence of DLS at baseline  Bruch’s membrane on horizontal B-scan  Bruch’s membrane on vertical B-scan  Bruch’s membrane at baseline | SFA horizontal  SFA vertical  SFA  CMT horizontal  CMT vertical  CMT  RNEL horizontal  RNEL vertical  RNEL  SRF horizontal  SRF vertical  SRF  ChT horizontal  ChT vertical  ChT  ChT (1-mo - B) horizontal  ChT (1-mo - B) vertical  ChT (1-mo - B)  EZ horizontal  EZ vertical  EZ  PED horizontal  PED vertical  PED  DLS horizontal  DLS vertical  DLS  Bruch’s membrane horizontal  Bruch’s membrane vertical  Bruch’s membrane | Subretinal fluid absorption on horizontal B-scan  Subretinal fluid absorption on vertical B-scan  Subretinal fluid absorption at 1-mo  CMT on horizontal B-scan  CMT on vertical B-scan  Average CMT at 1-mo  Thickness of RNEL on horizontal B-scan  Thickness of RNEL on vertical B-scan  Average thickness of RNEL at 1-mo  Height of SRF on horizontal B-scan  Height of SRF on vertical B-scan  Average height of SRF at 1-mo  ChT on horizontal B-scan  ChT on vertical B-scan  Average ChT at 1-mo  ChT variation (1-mo - baseline) on horizontal B-scan  ChT variation (1-mo - baseline) on vertical B-scan  Average ChT variation (1-mo - baseline) at 1-mo  Integrity of EZ on horizontal B-scan  Integrity of EZ on vertical B-scan  Average integrity of EZ at 1-mo  Existence of PED on horizontal B-scan  Existence of PED on vertical B-scan  Existence of PED at 1-mo  Existence of DLS on horizontal B-scan  Existence of DLS on vertical B-scan  Existence of DLS at 1-mo  Bruch’s membrane on horizontal B-scan  Bruch’s membrane on vertical B-scan  Bruch’s membrane at 1-mo | SFA horizontal  SFA vertical  SFA  CMT horizontal  CMT vertical  CMT  RNEL horizontal  RNEL vertical  RNEL  SRF horizontal  SRF vertical  SRF  ChT horizontal  ChT vertical  ChT  ChT (3-mo – 1-mo) horizontal  ChT (3-mo – 1-mo) vertical  ChT (3-mo – 1-mo)  EZ horizontal  EZ vertical  EZ  PED horizontal  PED vertical  PED  DLS horizontal  DLS vertical  DLS  Bruch’s membrane horizontal  Bruch’s membrane vertical  Bruch’s membrane  Recurrence horizontal  Recurrence vertical  Recurrence | Subretinal fluid absorption on horizontal B-scan  Subretinal fluid absorption on vertical B-scan  Subretinal fluid absorption at 3-mo  CMT on horizontal B-scan  CMT on vertical B-scan  Average CMT at 3-mo  Thickness of RNEL on horizontal B-scan  Thickness of RNEL on vertical B-scan  Average thickness of RNEL at 3-mo  Height of SRF on horizontal B-scan  Height of SRF on vertical B-scan  Average height of SRF at 3-mo  ChT on horizontal B-scan  ChT on vertical B-scan  Average ChT at 3-mo  ChT variation (3-mo – 1-mo) on horizontal B-scan  ChT variation (3-mo – 1-mo) on vertical B-scan  Average ChT variation (3-mo – 1-mo) at 3-mo  Integrity of EZ on horizontal B-scan  Integrity of EZ on vertical B-scan  Average integrity of EZ at 3-mo  Existence of PED on horizontal B-scan  Existence of PED on vertical B-scan  Existence of PED at 3-mo  Existence of DLS on horizontal B-scan  Existence of DLS on vertical B-scan  Existence of DLS at 3-mo  Bruch’s membrane on horizontal B-scan  Bruch’s membrane on vertical B-scan  Bruch’s membrane at 3-mo  Recurrence on horizontal B-scan  Recurrence on vertical B-scan  Recurrence at 3-mo | SFA horizontal  SFA vertical  SFA  CMT horizontal  CMT vertical  CMT  RNEL horizontal  RNEL vertical  RNEL  SRF horizontal  SRF vertical  SRF  ChT horizontal  ChT vertical  ChT  ChT (6-mo – 3-mo) horizontal  ChT (6-mo – 3-mo) vertical  ChT (6-mo – 3-mo)  EZ horizontal  EZ vertical  EZ  PED horizontal  PED vertical  PED  DLS horizontal  DLS vertical  DLS  Bruch’s membrane horizontal  Bruch’s membrane vertical  Bruch’s membrane  Recurrence horizontal  Recurrence vertical  Recurrence | Subretinal fluid absorption on horizontal B-scan  Subretinal fluid absorption on vertical B-scan  Subretinal fluid absorption at 6-mo  CMT on horizontal B-scan  CMT on vertical B-scan  Average CMT at 6-mo  Thickness of RNEL on horizontal B-scan  Thickness of RNEL on vertical B-scan  Average thickness of RNEL at 6-mo  Height of SRF on horizontal B-scan  Height of SRF on vertical B-scan  Average height of SRF at 6-mo  ChT on horizontal B-scan  ChT on vertical B-scan  Average ChT at 6-mo  ChT variation (6-mo – 3-mo) on horizontal B-scan  ChT variation (6-mo – 3-mo) on vertical B-scan  Average ChT variation (6-mo – 3-mo) at 6-mo  Integrity of EZ on horizontal B-scan  Integrity of EZ on vertical B-scan  Average integrity of EZ at 6-mo  Existence of PED on horizontal B-scan  Existence of PED on vertical B-scan  Existence of PED at 6-mo  Existence of DLS on horizontal B-scan  Existence of DLS on vertical B-scan  Existence of DLS at 6-mo  Bruch’s membrane on horizontal B-scan  Bruch’s membrane on vertical B-scan  Bruch’s membrane at 6-mo  Recurrence on horizontal B-scan  Recurrence on vertical B-scan  Recurrence at 6-mo |

**Table S1. Clinical Records and Imaging Features Used to Predict Visual Acuity**

This table shows all 20 clinical records and 145 imaging features used to predict VA. Twenty records (e.g., duration) were retrieved from the electronic medical records, 5 features (e.g., position and area of the leakage point) were calculated from FFA, 5 features (e.g., hyperperfusion and hypoperfusion) were calculated from ICGA, 12 features (e.g., the existence of abnormal reflection and branching vascular network [BVN]) were calculated from OCTA and 123 features (e.g., RNEL, CMT, and EZ) were calculated from OCT. Please see Figure 1 for a visualization of the measured features. VA, visual acuity; OCT, optical coherence tomography; OCTA, optical coherence tomography angiography; CSC, central serous chorioretinopathy; CL, conventional laser; SML, subthreshold micropulse laser; hd-PDT, half-dose photodynamic therapy; FFA, fundus fluorescein angiography; Single or multiple, a label of 1 indicates the existence of a single leakage point, and 2 indicates multiple leakage sites; Morphology, a label of 1 indicates smokestack leakage on FFA, 2 indicates focal diffuse leakage, and 3 indicates multiple diffuse leakage sites; Area, a label of 1 indicates that the area of leakage on FFA was less than the area of the optic disc, and 2 indicates a larger area; Position (position 1 to position 9), a label of 1 indicates that the damage was located less than 1500 microns away from the fovea, and 2 indicates a distance greater than 1500 microns; ICGA, indocyanine green angiography; High permeability, a label of 1 indicates the existence of high permeability, and 2 indicates normal permeability; Low permeability, a label of 1 indicates the existence of low permeability, and 2 indicates normal permeability; High reflection, a label of 1 indicates the existence of high reflection on OCTA, and 2 indicates normal reflection; Low reflection, a label of 1 indicates the existence of low reflection on OCTA, and 2 indicates normal reflection; BVN, a label of 1 indicates the existence of BVN, and 2 indicates a normal structure. All OCTA features are derived from images of the superficial choroidal layer, defined as 10 microns above the Bruch’s membrane to 30 microns below Bruch’s membrane in the 3*3 scanning pattern of Optovue (version 2017.1.0.155) software. SRF, subretinal fluid; CMT, central macular thickness; RNEL, retinal neuroepithelial layer; ChT, choroidal thickness, all measurements are expressed in microns; SFA, subretinal fluid absorption, a label of 1 indicates an increase or persistence in the level of unabsorbed SRF, 2 indicates partially absorbed SRF, and 3 indicates completely absorbed SRF; EZ, ellipsoid zone, a label of 1 indicates the complete absence of the original neurosensory retinal detachment area, 2 indicates the intermittent existence of the original neurosensory retinal detachment area with less than half of the total length, 3 indicates the existence of most of the original neurosensory retinal detachment area, and 4 indicates the complete existence of the original neurosensory retinal detachment area; PED, retinal pigment epithelial detachment, a label of 1 indicates the existence of PED, and 2 indicates a normal structure; DLS, double-layer sign, a label of 1 indicates the existence of DLS, and 2 indicates a normal structure; Bruch's membrane, a label of 1 indicates the disruption of the Bruch's membrane, and 2 indicates a normal membrane; Recurrence, a label of 1 indicates the reappearance of SRF, and 2 indicates a normal structure on OCT (in the analysis of quantitative data, we used the mean values of horizontal and vertical B-scans on OCT; in the analysis of qualitative data, we used the worse value between the horizontal and vertical B-scans on OCT).

1. Maier W, Buller R, Philipp M, et al. The Hamilton Anxiety Scale: reliability, validity and sensitivity to change in anxiety and depressive disorders. J Affect Disord. 1988;14(1):61-68.

2. Manzar MD, BaHammam AS, Hameed UA, et al. Dimensionality of the Pittsburgh Sleep Quality Index: a systematic review. Health Qual Life Outcomes. 2018;16(1):89.

3. Yannuzzi LA. Type-A behavior and central serous chorioretinopathy. Retina. 1987;7(2):111-131

| **Clinical data** | | | | | | | |
| --- | --- | --- | --- | --- | --- | --- | --- |
| **Feature** | **Description** | **Feature** | **Description** | **Feature** | **Description** | **Feature** | **Description** |
| Age  Sex | Age of the patient  Sex of the patient | Duration  Therapy | Duration of CSC  CL, SML, or hd-PDT | VA Baseline  VA 1-mo | VA before treatment  VA at 1-mo after treatment | VA 3-mo  VA 6-mo | VA at 3-mo after treatment  VA at 6-mo after treatment |
| **Features from OCT (Baseline)** | | **Features from OCT (1-mo)** | | **Features from OCT (3-mo)** | | **Features from OCT (6-mo)** | |
| **Feature** | **Description** | **Feature** | **Description** | **Feature** | **Description** | **Feature** | **Description** |
| SFA horizontal  SFA vertical  SFA  CMT horizontal  CMT vertical  CMT  RNEL horizontal  RNEL vertical  RNEL  SRF horizontal  SRF vertical  SRF  ChT horizontal  ChT vertical  ChT  EZ horizontal  EZ vertical  EZ  PED horizontal  PED vertical  PED  DLS horizontal  DLS vertical  DLS  Bruch’s membrane horizontal  Bruch’s membrane vertical  Bruch’s membrane | Subretinal fluid absorption on horizontal B-scan  Subretinal fluid absorption on vertical B-scan  Subretinal fluid absorption at baseline  CMT on horizontal B-scan  CMT on vertical B-scan  Average CMT at baseline  Thickness of RNEL on horizontal B-scan  Thickness of RNEL on vertical B-scan  Average thickness of RNEL at baseline  Height of SRF on horizontal B-scan  Height of SRF on vertical B-scan  Average height of SRF at baseline  ChT on horizontal B-scan  ChT on vertical B-scan  Average ChT at baseline  Integrity of EZ on horizontal B-scan  Integrity of EZ on vertical B-scan  Average integrity of EZ at baseline  Existence of PED on horizontal B-scan  Existence of PED on vertical B-scan  Existence of PED at baseline  Existence of DLS on horizontal B-scan  Existence of DLS on vertical B-scan  Existence of DLS at baseline  Bruch’s membrane on horizontal B-scan  Bruch’s membrane on vertical B-scan  Bruch’s membrane at baseline | SFA horizontal  SFA vertical  SFA  CMT horizontal  CMT vertical  CMT  RNEL horizontal  RNEL vertical  RNEL  SRF horizontal  SRF vertical  SRF  ChT horizontal  ChT vertical  ChT  ChT (1-mo - B) horizontal  ChT (1-mo - B) vertical  ChT (1-mo - B)  EZ horizontal  EZ vertical  EZ  PED horizontal  PED vertical  PED  DLS horizontal  DLS vertical  DLS  Bruch’s membrane horizontal  Bruch’s membrane vertical  Bruch’s membrane | Subretinal fluid absorption on horizontal B-scan  Subretinal fluid absorption on vertical B-scan  Subretinal fluid absorption at 1-mo  CMT on horizontal B-scan  CMT on vertical B-scan  Average CMT at 1-mo  Thickness of RNEL on horizontal B-scan  Thickness of RNEL on vertical B-scan  Average thickness of RNEL at 1-mo  Height of SRF on horizontal B-scan  Height of SRF on vertical B-scan  Average height of SRF at 1-mo  ChT on horizontal B-scan  ChT on vertical B-scan  Average ChT at 1-mo  ChT variation (1-mo - baseline) on horizontal B-scan  ChT variation (1-mo - baseline) on vertical B-scan  Average ChT variation (1-mo - baseline) at 1-mo  Integrity of EZ on horizontal B-scan  Integrity of EZ on vertical B-scan  Average integrity of EZ at 1-mo  Existence of PED on horizontal B-scan  Existence of PED on vertical B-scan  Existence of PED at 1-mo  Existence of DLS on horizontal B-scan  Existence of DLS on vertical B-scan  Existence of DLS at 1-mo  Bruch’s membrane on horizontal B-scan  Bruch’s membrane on vertical B-scan  Bruch’s membrane at 1-mo | SFA horizontal  SFA vertical  SFA  CMT horizontal  CMT vertical  CMT  RNEL horizontal  RNEL vertical  RNEL  SRF horizontal  SRF vertical  SRF  ChT horizontal  ChT vertical  ChT  ChT (3-mo – 1-mo) horizontal  ChT (3-mo – 1-mo) vertical  ChT (3-mo – 1-mo)  EZ horizontal  EZ vertical  EZ  PED horizontal  PED vertical  PED  DLS horizontal  DLS vertical  DLS  Bruch’s membrane horizontal  Bruch’s membrane vertical  Bruch’s membrane  Recurrence horizontal  Recurrence vertical  Recurrence | Subretinal fluid absorption on horizontal B-scan  Subretinal fluid absorption on vertical B-scan  Subretinal fluid absorption at 3-mo  CMT on horizontal B-scan  CMT on vertical B-scan  Average CMT at 3-mo  Thickness of RNEL on horizontal B-scan  Thickness of RNEL on vertical B-scan  Average thickness of RNEL at 3-mo  Height of SRF on horizontal B-scan  Height of SRF on vertical B-scan  Average height of SRF at 3-mo  ChT on horizontal B-scan  ChT on vertical B-scan  Average ChT at 3-mo  ChT variation (3-mo – 1-mo) on horizontal B-scan  ChT variation (3-mo – 1-mo) on vertical B-scan  Average ChT variation (3-mo – 1-mo) at 3-mo  Integrity of EZ on horizontal B-scan  Integrity of EZ on vertical B-scan  Average integrity of EZ at 3-mo  Existence of PED on horizontal B-scan  Existence of PED on vertical B-scan  Existence of PED at 3-mo  Existence of DLS on horizontal B-scan  Existence of DLS on vertical B-scan  Existence of DLS at 3-mo  Bruch’s membrane on horizontal B-scan  Bruch’s membrane on vertical B-scan  Bruch’s membrane at 3-mo  Recurrence on horizontal B-scan  Recurrence on vertical B-scan  Recurrence at 3-mo | SFA horizontal  SFA vertical  SFA  CMT horizontal  CMT vertical  CMT  RNEL horizontal  RNEL vertical  RNEL  SRF horizontal  SRF vertical  SRF  ChT horizontal  ChT vertical  ChT  ChT (6-mo – 3-mo) horizontal  ChT (6-mo – 3-mo) vertical  ChT (6-mo – 3-mo)  EZ horizontal  EZ vertical  EZ  PED horizontal  PED vertical  PED  DLS horizontal  DLS vertical  DLS  Bruch’s membrane horizontal  Bruch’s membrane vertical  Bruch’s membrane  Recurrence horizontal  Recurrence vertical  Recurrence | Subretinal fluid absorption on horizontal B-scan  Subretinal fluid absorption on vertical B-scan  Subretinal fluid absorption at 6-mo  CMT on horizontal B-scan  CMT on vertical B-scan  Average CMT at 6-mo  Thickness of RNEL on horizontal B-scan  Thickness of RNEL on vertical B-scan  Average thickness of RNEL at 6-mo  Height of SRF on horizontal B-scan  Height of SRF on vertical B-scan  Average height of SRF at 6-mo  ChT on horizontal B-scan  ChT on vertical B-scan  Average ChT at 6-mo  ChT variation (6-mo – 3-mo) on horizontal B-scan  ChT variation (6-mo – 3-mo) on vertical B-scan  Average ChT variation (6-mo – 3-mo) at 6-mo  Integrity of EZ on horizontal B-scan  Integrity of EZ on vertical B-scan  Average integrity of EZ at 6-mo  Existence of PED on horizontal B-scan  Existence of PED on vertical B-scan  Existence of PED at 6-mo  Existence of DLS on horizontal B-scan  Existence of DLS on vertical B-scan  Existence of DLS at 6-mo  Bruch’s membrane on horizontal B-scan  Bruch’s membrane on vertical B-scan  Bruch’s membrane at 6-mo  Recurrence on horizontal B-scan  Recurrence on vertical B-scan  Recurrence at 6-mo |

**Table S2. Clinical Records and Imaging Features Used to Predict Visual Acuity in Simplified Model Ⅰ**

This table shows all 8 clinical records and 123 imaging features used to predict VA in model Ⅰ. Eight records (e.g., duration) were retrieved from the electronic medical records, and 123 features (e.g., RNEL, CMT, and EZ) were calculated from OCT imaging. VA, visual acuity; OCT, optical coherence tomography; CSC, central serous chorioretinopathy; CL, conventional laser; SML, subthreshold micropulse laser; hd-PDT, half-dose photodynamic therapy; SRF, subretinal fluid; CMT, central macular thickness; RNEL, retinal neuroepithelial layer; ChT, choroidal thickness, all measurements are expressed in microns; SFA, subretinal fluid absorption, a label of 1 indicates an increase or persistence in the level of unabsorbed SRF, 2 indicates partially absorbed SRF, and 3 indicates completely absorbed SRF; EZ, ellipsoid zone, a label of 1 indicates the complete absence of the original neurosensory retinal detachment area, 2 indicates the intermittent existence of the original neurosensory retinal detachment area with less than half of the total length, 3 indicates the existence of most of the original neurosensory retinal detachment area, and 4 indicates the complete existence of the original neurosensory retinal detachment area; PED, retinal pigment epithelial detachment, a label of 1 indicates the existence of PED, and 2 indicates a normal structure; DLS, double-layer sign, a label of 1 indicates the existence of DLS, and 2 indicates a normal structure; Bruch's membrane, a label of 1 indicates the disruption of the Bruch's membrane, and 2 indicates a normal membrane; Recurrence, a label of 1 indicates the reappearance of SRF, and 2 indicates a normal structure on OCT (in the analysis of quantitative data, we used the mean values of horizontal and vertical B-scans on OCT; in the analysis of qualitative data, we used the worse value of the horizontal and vertical B-scans on OCT).

1. Maier W, Buller R, Philipp M, et al. The Hamilton Anxiety Scale: reliability, validity and sensitivity to change in anxiety and depressive disorders. J Affect Disord. 1988;14(1):61-68.

2. Manzar MD, BaHammam AS, Hameed UA, et al. Dimensionality of the Pittsburgh Sleep Quality Index: a systematic review. Health Qual Life Outcomes. 2018;16(1):89.

**Table S3. Accuracy of Visual Acuity Prediction with Additional Previous Data Using the Full Model**

| **Algorithm Learner** | **3-mo** | | **6-mo** | | **6-mo** | |
| --- | --- | --- | --- | --- | --- | --- |
| **Databases** | **Baseline + 1-mo** | | **Baseline + 1-mo** | | **Baseline + 1-mo + 3-mo** | |
| **Validation Set** | **MAE** | **RMSE** | **MAE** | **RMSE** | **MAE** | **RMSE** |
| LASSO | 0.060 ± 0.011 | 0.080 ± 0.019* | 0.071 ± 0.010 | 0.093 ± 0.013 | 0.064 ± 0.010 | 0.088 ± 0.014 |
| AdaBoost | 0.074 ± 0.010 | 0.100 ± 0.020 | 0.075 ± 0.010 | 0.102 ± 0.018 | 0.072 ± 0.010 | 0.099 ± 0.017 |
| Gradient Boosting | 0.068 ± 0.011 | 0.095 ± 0.021 | 0.072 ± 0.010 | 0.100 ± 0.016 | 0.072 ± 0.010 | 0.103 ± 0.018 |
| XGBoost | 0.066 ± 0.011 | 0.090 ± 0.022 | 0.072 ± 0.010 | 0.095 ± 0.015* | 0.070 ± 0.008 | 0.100 ± 0.013 |
| Random Forest | 0.067 ± 0.010 | 0.097 ± 0.019 | 0.070 ± 0.010 | 0.100 ± 0.018 | 0.067 ± 0.008 | 0.097 ± 0.016 |
| Extra-Trees | 0.068 ± 0.014 | 0.097 ± 0.026 | 0.073 ± 0.009 | 0.103 ± 0.015 | 0.070 ± 0.007 | 0.099 ± 0.013 |
| Blending Algorithm | 0.060 ± 0.011* | 0.082 ± 0.022 | 0.066 ± 0.010* | 0.091 ± 0.015 | 0.062 ± 0.008* | 0.089 ± 0.013* |

MAE, mean absolute error; RMSE, root mean square error; XEC, Xiamen Eye Center. Accuracy (VA in logMAR) of VA prediction at 3 and 6 months after laser treatment compared with the ground truth. The results were stratified according to the follow-up period and the points input into the algorithms; this table shows the predictive effect of the baseline data and one or more follow-up datasets. All VA predictions are shown with the standard deviation (in logMAR). The best predictions have been marked with asterisks in the validation set.

**Table S4. Accuracy of Visual Acuity Prediction with Additional Previous Data Using Simplified Model Ⅰ**

| **Algorithm Learner** | **3-mo** | | **6-mo** | | **6-mo** | |
| --- | --- | --- | --- | --- | --- | --- |
| **Databases** | **Baseline + 1-mo** | | **Baseline + 1-mo** | | **Baseline + 1-mo + 3-mo** | |
| **Validation Set** | **MAE** | **RMSE** | **MAE** | **RMSE** | **MAE** | **RMSE** |
| LASSO | 0.059 ± 0.012 | 0.079 ± 0.021* | 0.069 ± 0.010 | 0.094 ± 0.015* | 0.064 ± 0.008 | 0.089 ± 0.014 |
| AdaBoost | 0.076 ± 0.010 | 0.102 ± 0.020 | 0.082 ± 0.011 | 0.108 ± 0.013 | 0.079 ± 0.010 | 0.108 ± 0.015 |
| Gradient Boosting | 0.066 ± 0.011 | 0.094 ± 0.019 | 0.081 ± 0.009 | 0.115 ± 0.015 | 0.078 ± 0.009 | 0.111 ± 0.016 |
| XGBoost | 0.066 ± 0.012 | 0.090 ± 0.020 | 0.074 ± 0.010 | 0.105 ± 0.016 | 0.073 ± 0.008 | 0.103 ± 0.014 |
| Random Forest | 0.066 ± 0.011 | 0.095 ± 0.021 | 0.072 ± 0.010 | 0.101 ± 0.016 | 0.069 ± 0.008* | 0.099 ± 0.014 |
| Extra-Trees | 0.068 ± 0.014 | 0.096 ± 0.025 | 0.072 ± 0.009 | 0.106 ± 0.016 | 0.070 ± 0.008 | 0.099 ± 0.015 |
| Blending Algorithm | 0.059 ± 0.012* | 0.082 ± 0.022 | 0.067 ± 0.010* | 0.094 ± 0.015* | 0.064 ± 0.007* | 0.091 ± 0.012* |
| **XEC Set** | **MAE** | **RMSE** | **MAE** | **RMSE** | **MAE** | **RMSE** |
| Blending Algorithm | 0.063 (0.043-0.089) | 0.091 (0.058-0.120) | 0.093 (0.064-0.127) | 0.118 (0.079-0.160) | 0.089 (0.067-0.112) | 0.104 (0.079-0.127) |

MAE, mean absolute error; RMSE, root mean square error; XEC, Xiamen Eye Center. Accuracy (VA in logMAR) of VA prediction at 3 and 6 months after laser treatment compared with the ground truth. The results were stratified according to the follow-up period and the points input into the algorithms; this table shows the predictive effect of the baseline data and one or more follow-up datasets. All VA predictions are shown with the standard deviation (in logMAR). The best predictions have been marked with asterisks in the validation set.

**Table S5. Accuracy of Visual Acuity Prediction with Additional Previous Data Using Simplified Model Ⅱ**

| **Algorithm Learner** | **3-mo** | | **6-mo** | | **6-mo** | |
| --- | --- | --- | --- | --- | --- | --- |
| **Databases** | **Baseline + 1-mo** | | **Baseline + 1-mo** | | **Baseline + 1-mo + 3-mo** | |
| **Validation Set** | **MAE** | **RMSE** | **MAE** | **RMSE** | **MAE** | **RMSE** |
| LASSO | 0.060 ± 0.010* | 0.078 ± 0.018* | 0.067 ± 0.010* | 0.093 ± 0.015* | 0.062 ± 0.009* | 0.088 ± 0.014* |
| AdaBoost | 0.078 ± 0.011 | 0.102 ± 0.019 | 0.085 ± 0.006 | 0.110 ± 0.012 | 0.083 ± 0.012 | 0.110 ± 0.016 |
| Gradient Boosting | 0.067 ± 0.013 | 0.093 ± 0.022 | 0.088 ± 0.011 | 0.120 ± 0.018 | 0.082 ± 0.011 | 0.115 ± 0.019 |
| XGBoost | 0.067 ± 0.010 | 0.094 ± 0.019 | 0.078 ± 0.013 | 0.108 ± 0.019 | 0.076 ± 0.009 | 0.104 ± 0.015 |
| Random Forest | 0.066 ± 0.011 | 0.095 ± 0.020 | 0.075 ± 0.009 | 0.104 ± 0.015 | 0.072 ± 0.007 | 0.102 ± 0.014 |
| Extra-Trees | 0.065 ± 0.012 | 0.092 ± 0.021 | 0.071 ± 0.008 | 0.098 ± 0.013 | 0.070 ± 0.008 | 0.098 ± 0.013 |
| Blending Algorithm | 0.060 ± 0.011 | 0.082 ± 0.021 | 0.068 ± 0.009 | 0.094 ± 0.013 | 0.065 ± 0.007 | 0.091 ± 0.011 |
| **XEC Set** | **MAE** | **RMSE** | **MAE** | **RMSE** | **MAE** | **RMSE** |
| Blending Algorithm | 0.066 (0.044-0.090) | 0.094 (0.060-0.122) | 0.101 (0.070-0.136) | 0.127 (0.082-0.171) | 0.090 (0.067-0.117) | 0.107 (0.074-0.136) |

MAE, mean absolute error; RMSE, root mean square error; XEC, Xiamen Eye Center. Accuracy (VA in logMAR) of VA prediction at 3 and 6 months after laser treatment compared with the ground truth. The results were stratified according to the follow-up period and the points input into the algorithms; this table shows the predictive effect of the baseline data and one or more follow-up datasets. All VA predictions are shown with the standard deviation (in logMAR). The best predictions have been marked with asterisks in the validation set.

**Table S6. Accuracy of Visual Acuity Prediction with Additional Previous Data Using Simplified Model Ⅲ**

| **Algorithm Learner** | **3-mo** | | **6-mo** | | **6-mo** | |
| --- | --- | --- | --- | --- | --- | --- |
| **Databases** | **Baseline + 1-mo** | | **Baseline + 1-mo** | | **Baseline + 1-mo + 3-mo** | |
| **Validation Set** | **MAE** | **RMSE** | **MAE** | **RMSE** | **MAE** | **RMSE** |
| LASSO | 0.080 ± 0.011 | 0.112 ± 0.021 | 0.081 ± 0.009 | 0.113 ± 0.023 | 0.079 ± 0.011 | 0.110 ± 0.021 |
| AdaBoost | 0.086 ± 0.010 | 0.115 ± 0.017 | 0.087 ± 0.006 | 0.114 ± 0.013 | 0.086 ± 0.005 | 0.116 ± 0.014 |
| Gradient Boosting | 0.084 ± 0.012 | 0.114 ± 0.020 | 0.090 ± 0.014 | 0.120 ± 0.018 | 0.089 ± 0.012 | 0.119 ± 0.014 |
| XGBoost | 0.078 ± 0.008 | 0.109 ± 0.018 | 0.083 ± 0.008 | 0.112 ± 0.012 | 0.081 ± 0.009 | 0.110 ± 0.013 |
| Random Forest | 0.078 ± 0.009 | 0.109 ± 0.018 | 0.081 ± 0.008 | 0.110 ± 0.013 | 0.078 ± 0.008 | 0.108 ± 0.013 |
| Extra-Trees | 0.078 ± 0.014 | 0.109 ± 0.024 | 0.075 ± 0.006 | 0.103 ± 0.016 | 0.075 ± 0.009 | 0.104 ± 0.015 |
| Blending Algorithm | 0.076 ± 0.010* | 0.106 ± 0.020* | 0.075 ± 0.007* | 0.105 ± 0.017* | 0.074 ± 0.008* | 0.104 ± 0.016* |
| **XEC Set** | **MAE** | **RMSE** | **MAE** | **RMSE** | **MAE** | **RMSE** |
| Blending Algorithm | 0.087 (0.064-0.113) | 0.114 (0.078-0.148) | 0.109 (0.073-0.149) | 0.140 (0.087-0.190) | 0.103 (0.067-0.142) | 0.132 (0.087-0.181) |

MAE, mean absolute error; RMSE, root mean square error; XEC, Xiamen Eye Center. Accuracy (VA in logMAR) of VA prediction at 3 and 6 months after laser treatment compared with the ground truth. The results were stratified according to the follow-up period and the points input into the algorithms; this table shows the predictive effect of the baseline data and one or more follow-up datasets. All VA predictions are shown with the standard deviation (in logMAR). The best predictions have been marked with asterisks in the validation set.

**Figure S1. Features Measured in Images**


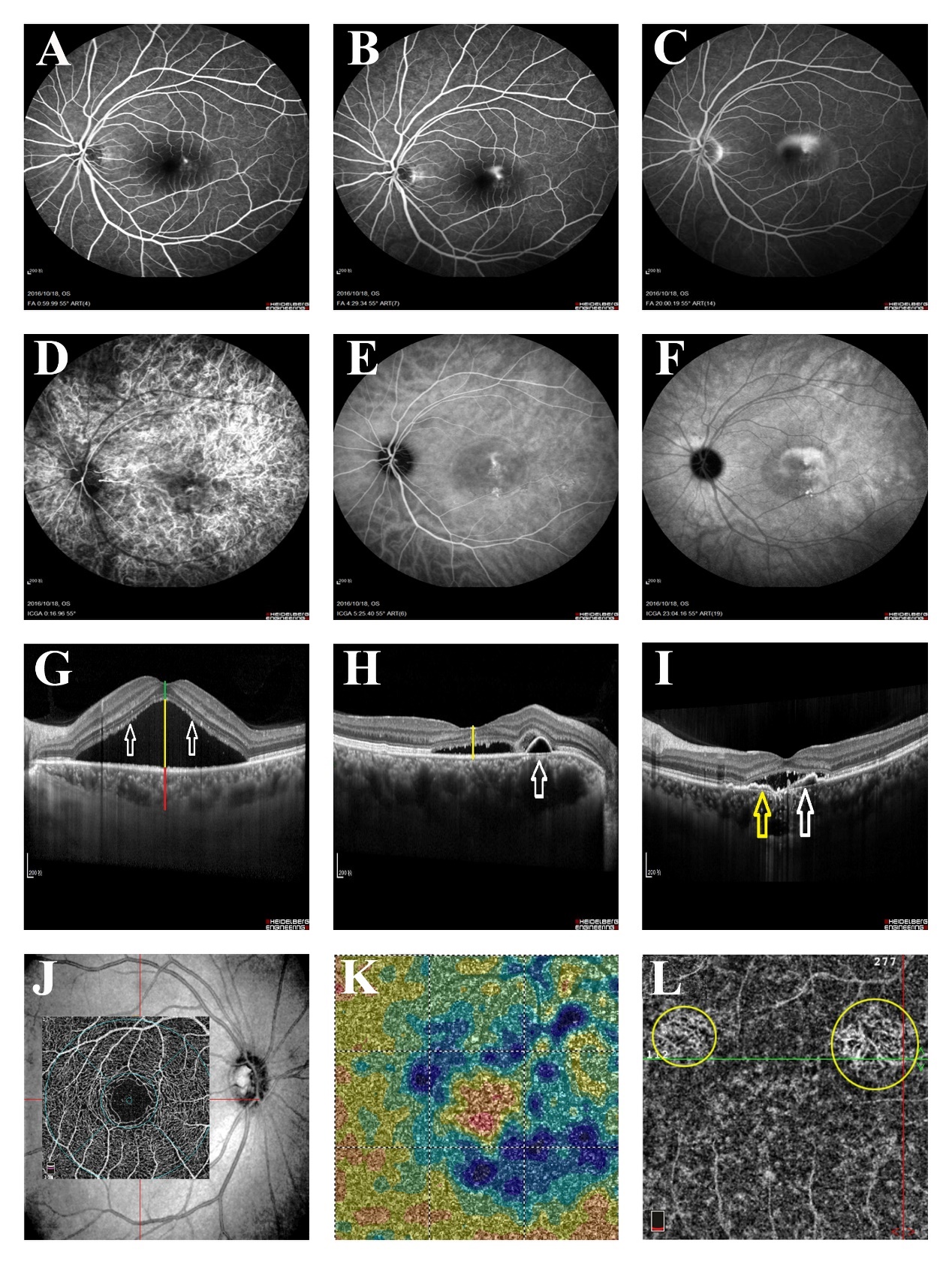


All imaging features used to predict VA. Please see Table S1 for a detailed description of all measured features. (A, B, and C) Early, middle and late phase FFA of the left eye of a 46-year-old patient with CSC. (D, E, and F) Contemporaneous ICGA of the same patient. (G) Horizontal B-scan OCT of a patient with CSC, and manual measurements are labeled as follows: green line, RNEL; yellow line, SRF; and red line, ChT; (H) Yellow line, CMT; White arrow, PED; and (I) Yellow arrow, DLS; White arrow, Bruch's membrane. (J) The en face projection slab area of the 3*3 pattern on OCTA. (K) An area of high reflection related to active leakage and low reflection was surrounded in the superficial choroidal layer. (L) The observation of a superficial choroidal layer on OCTA confirmed the presence of BVN. FFA, fundus fluorescein angiography; ICGA, indocyanine green angiography; OCT, optical coherence tomography; OCTA, optical coherence tomography angiography; SRF, subretinal fluid; RNEL, retinal neuroepithelial layer; ChT, choroidal thickness; PED, retinal pigment epithelial detachment; DLS, double-layer sign; BVN, branching vascular network.

**Figure S2. Visual Acuity Prediction Fitting Curve of Simplified Model Ⅰ**


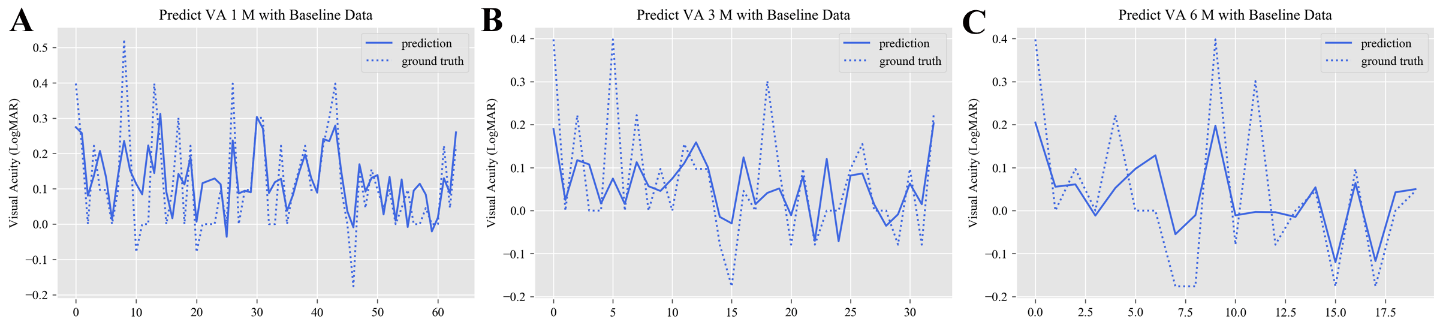


These plots show the differences between the VA predictions (solid line) and the ground truth (dotted line) based on the XEC set with simplified model Ⅰ. The x-axis represents individual eyes of patients as consecutive numbers. The y-axis shows VA (in logarithm of minimum angle of resolution [logMAR] units). VA, visual acuity; XEC, Xiamen Eye Center.

**Figure S3. Visual Acuity Prediction Fitting Curve of Simplified Model** **Ⅱ**


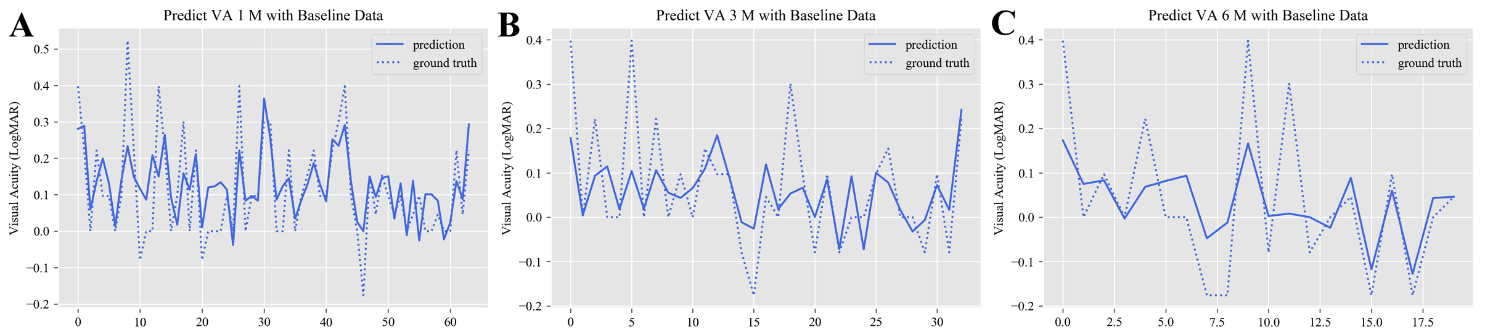


These plots show the differences between the VA predictions (solid line) and the ground truth (dotted line) based on the XEC set with simplified model Ⅱ. The x-axis represents individual eyes of patients as consecutive numbers. The y-axis shows VA (in logarithm of minimum angle of resolution [logMAR] units). VA, visual acuity; XEC, Xiamen Eye Center.

**Figure S4. Visual Acuity Prediction Fitting Curve of Simplified Model Ⅲ**


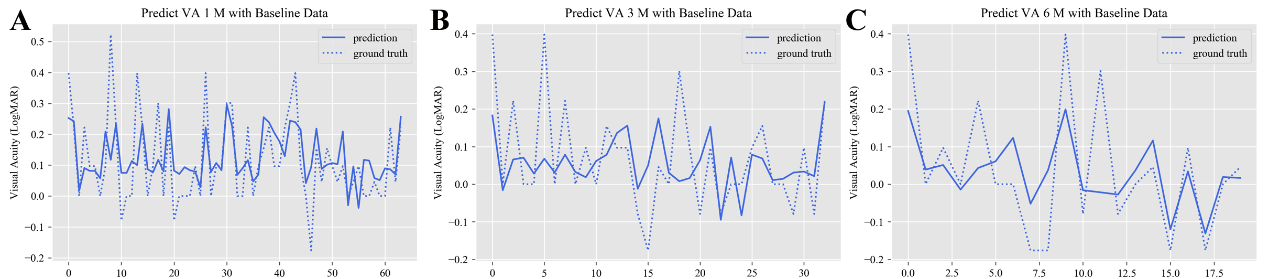


These plots show the differences between the VA predictions (solid line) and the ground truth (dotted line) based on the XEC set with simplified model Ⅲ. The x-axis represents individual eyes of patients as consecutive numbers. The y-axis shows VA (in logarithm of minimum angle of resolution [logMAR] units). VA, visual acuity; XEC, Xiamen Eye Center.
